# Effects of administering berberine alone or in combination on type 2 diabetes mellitus: a systematic review and meta-analysis

**DOI:** 10.3389/fphar.2024.1455534

**Published:** 2024-11-21

**Authors:** Jiacheng Wang, Chenhao Bi, Hongbin Xi, Fengqin Wei

**Affiliations:** ^1^ College of Traditional Chinese Medicine, Shandong University of Traditional Chinese Medicine, Jinan, China; ^2^ Department of Traditional Chinese Medicine Classics, Tai’an Hospital of Traditional Chinese Medicine, Tai’an, China

**Keywords:** berberine, type 2 diabetes mellitus, traditional Chinese medicine, safety, meta-analysis

## Abstract

**Background:**

Despite the availability of multiple therapies for Type 2 diabetes mellitus (T2DM), challenges remain due to side effects and efficacy limitations. Berberine (BBR) has shown broad anti-diabetic effects, prompting a systematic assessment of its efficacy and safety through a meta-analysis.

**Methods:**

A comprehensive search was conducted across eight database and search engines from inception until 06/09/2024. Only randomized controlled trials (RCTs) meeting inclusion criteria were analyzed. The Cochrane risk of bias assessment tool and Jadad scale were used to evaluate study quality. Meta-analysis was performed using RevMan v5.3 and Stata/SE v15.1.

**Results:**

Fifty studies involving 4,150 participants were included. BBR alone significantly reduced fasting plasma glucose (FPG) (MD = −0.59 mmol/L, *p* = 0.048), 2-h postprandial blood glucose (2hPBG) (MD = −1.57 mmol/L, *p* < 0.01), low-density lipoprotein cholesterol (LDL-C) (MD = −0.30 mmol/L, *p* < 0.01), total cholesterol (TC) (MD = −0.30 mmol/L, *p* = 0.034), and triglycerides (TG) (MD = −0.35 mmol/L, *p* < 0.01). When combined with hypoglycemic drugs, BBR significantly improved FPG (MD = −0.99 mmol/L, *p* < 0.01), 2hPBG (MD = −1.07 mmol/L, *p* < 0.01), glycated hemoglobin (HbA1c) (MD = −0.69%, *p* < 0.01), and other metabolic markers, including fasting insulin (Fins), homeostasis model assessment index for assessing insulin resistance (HOMA-IR), lipid profiles and inflammatory markers. The most common BBR dosage was 0.9–1.5 g/d, with treatment cycles typically lasting 1–3 months.

**Conclusion:**

Current evidence suggests that BBR alone or in combination has significant potential for treating type 2 diabetes mellitus (T2DM). Future research should encompass a broader scope, including not just the beneficial effects of BBR in head-to-head studies, but more crucially, delving into its mechanisms of action with hypoglycemic drugs to optimize T2DM treatment strategies.

## 1 Introduction

As per the International Diabetes Federation (IDF), the global prevalence of diabetes mellitus (DM) among individuals aged 20–79 was estimated to be approximately 537 million in 2021, accounting for 10.5% of the global population. This figure is projected to increase to 643 million (11.3%) by 2030 and further to 783 million (12.2%) by 2045 (Sun et al., 2022). Among these cases, more than 90% are attributed to type 2 diabetes (T2DM) (Fan et al., 2024). T2DM is recognized as a chronic metabolic disorder characterized by hyperglycemia, insulin resistance, and dyslipidemia (American Diabetes Association Professional Practice Committee, 2024). Complications of T2DM, including various microvascular and macrovascular issues, severely impact patients’ quality of life and may lead to organ dysfunction and even death (Ahmad et al., 2022). Studies have shown that the risk of premature death in patients with T2DM is approximately 15% higher compared to the general population (Tancredi et al., 2015). Moreover, The impact of T2DM is further evidenced by the rising age-standardized mortality rates and the increasing disability-adjusted life years (DALYs), coupled with the substantial healthcare costs, particularly in high-income nations, posing a significant burden on the socioeconomic fabric and the quality of life of the population (Safiri et al., 2022; Ye et al., 2023; Butt et al., 2024). Consequently, there is an urgent need for more effective and safer strategies for prevention and treatment.

Conventional hypoglycemic agents administered orally, such as Metformin (Met), thiazolidinediones (TZDs), sulfonylureas (SU), and α-glucosidase inhibitors (AGI), are extensively utilized in the treatment of T2DM. However, they are associated with various adverse reactions, including gastrointestinal discomfort, hypoglycemia, and edema, as well as hepatic and renal impairment (Sanchez-Rangel and Inzucchi, 2017; Mehrpour et al., 2022; Derosa, 2010). Since the 21st century, a new class of hypoglycemic agents has emerged, agents like dipeptidyl peptidase-4 inhibitors (DPP-4i), glucagon-like peptide-1 receptor agonists (GLP-1RA), and sodium-glucose cotransporter-2 inhibitors (SGLT-2i), have been marketed. While these drugs exhibit promising outcomes in blood glucose control and complication reduction, they still harbor limitations and potential risks (Douros et al., 2020; Li et al., 2016). For instance, The osmotic diuretic effect of SGLT-2 inhibitors may lead to decreased blood volume and tissue perfusion, consequently increasing the risk of lower extremity complications, including amputations (Potier et al., 2021; Xu et al., 2022; Yang T. et al., 2023); patients treated with DPP-4 inhibitors face a heightened incidence of cardiovascular events and adverse renal outcomes (Giugliano et al., 2022); GLP-1RA administration is intricate and may result in weight rebound post-discontinuation, significantly impacting patient tolerance and long-term compliance (Drucker et al., 2008; Weiss et al., 2022). Moreover, the high cost of these new hypoglycemic agents restricts their accessibility and affordability in certain regions (Ansari et al., 2022). Noteworthily, more researchers are turning to plant-derived traditional herbal medicines due to their significant efficacy, holistic regulation, and generally higher safety (Jovanovski et al., 2021; Samad et al., 2017; Zhang Y. et al., 2024).

Berberine (BBR), also known as Huangliansu, is a yellow crystalline isoquinoline alkaloid found in the roots, stems, leaves, and fruits of plants from the Ranunculaceae, Berberidaceae, Menispermaceae, Papaveraceae, and Rutaceae families (Neag et al., 2018). The Ranunculaceae herb Coptis chinensis has the highest BBR content, measured by high-performance liquid chromatography (HPLC) to be between 51.14 and 96.10 mg/g (Shrivastava et al., 2023). As a traditional medicine with a long history, BBR has been widely used to treat metabolic diseases, such as obesity, polycystic ovary syndrome, hyperlipidemia, coronary artery disease, and gout (Song et al., 2020). In the treatment of T2DM, although certain herbal therapies, such as okra, have shown certain potential, existing studies have demonstrated their limitations in reducing HbA1c (Mokgalaboni et al., 2023). In contrast, BBR not only significantly ameliorates glycemic control but also intervenes in the pathological state of T2DM through multiple molecular mechanisms, including improving insulin resistance, regulating gut microbiota, and exerting anti-inflammatory and antioxidant effects (Kong et al., 2009; He et al., 2022; Utami et al., 2023). These features effectively address the limitations of existing treatment options and demonstrate promise as a new first-line therapeutic agent. Additionally, current systematic reviews have predominantly focused on BBR’s effectiveness in modulating blood glucose (Xie et al., 2022; Lan et al., 2015; Dong et al., 2012; Liang et al., 2019). Its holistic influence on glucose metabolism, insulin secretion, and lipid profiles in patients with T2DM remains incompletely validated. Thus, this study was conducted to perform a meta-analysis to thoroughly investigate the overall effects of BBR, either as a monotherapy or in combination with other hypoglycemic agents, on patients with T2DM, with the aim of providing insights and evidence for optimizing clinical treatment strategies.

## 2 Methods

Adhering to the PRISMA guidelines (Page et al., 2021), this study was conducted and registered with PROSPERO (ID: CRD42024519428).

### 2.1 Literature search

The search period spanned from the inception of database and search engines to 06/09/2024. An extensive search was done across the PubMed, Embase, Cochrane Library, Web of Science, China National Knowledge Infrastructure Database (CNKI), Wan-Fang Database, Chinese Scientific Journals Full-text Database (VIP), and the Chinese Biomedical Literature Database (SinoMed) using the keywords: [“T2DM” or “Type 2 Diabetes Mellitus” or “insulin-independent diabetes mellitus” or “xiaokebing” or “tangniaobing”] AND [“Berberine” or “umbellatine” or “huangliansu”]. Detailed search strategies are available in Supplementary Table 1. Additionally, we manually reviewed the references of relevant articles to ensure that there was a thorough coverage of the literature.

### 2.2 Criteria for study inclusion/exclusion

#### 2.2.1 Inclusion criteria

Population: Patients diagnosed with T2DM as per the 1999 World Health Organization criteria or the “Chinese Guidelines for the Prevention and Treatment of Type 2 Diabetes Mellitus (2020 Edition)”.

Intervention: BBR administered either alone or in combination with other oral hypoglycemic agents or probiotics.

Comparison: Control group subjects receiving interventions other than BBR, such as placebo, oral hypoglycemic agents, or probiotics.

Outcome: Primary outcome measures: 1) Fasting plasma glucose (FPG); 2) glycated hemoglobin (HbA1c); 3) fasting insulin (Fins). Secondary outcome measures: 1) 2-h postprandial glucose (2hPBG); 2) homeostatic model assessment for insulin resistance (HOMA-IR); 3) lipid profiles (LDL-C, HDL-C, TC, TG); 4) inflammatory markers (CRP, IL-6, TNF-α); 5) adverse events. Included studies should report at least one primary outcome and may include one or more secondary outcome measures.

Design: Only RCTs were deemed eligible for this study.

#### 2.2.2 Exclusion criteria

Population: Studies involving patients with either acute or chronic complications, such as optic neuropathy, non-alcoholic fatty liver disease, urinary tract infections, hyperlipidemia, hypertension, cerebral infarction, peptic ulcer disease, and diabetic ketoacidosis.

Intervention: Interventions with BBR-containing nutritional supplements or other pharmaceutical preparations such as Berberis vulgaris root extract or dried stem powder of Berberis aristata; the hypoglycemic medications used in combination interventions or the control group were not explicitly reported.

Comparison: Control measures that did not assess the effect of BBR, e.g., BBR + Met vs BBR or BBR + Met vs placebo.

Outcome: Insufficiently detailed, inaccessible, or incomplete baseline data for key outcome measures.

Study design: Self-controlled studies, animal experiments, reviews, unpublished conference abstracts, or duplicates.

### 2.3 Literature search and data extraction

All search results were imported into EndNote X9 (Thomson Reuters, New York, NY, United States). Following the removal of duplicate entries, the preliminary screening entailed evaluating titles and abstracts was conducted to filter out studies that did not align with the predefined criteria. Subsequently, full-text articles underwent a thorough review to finalize inclusion. Data extraction from the included studies encompassed the following categories: 1) Basic information: title, first author, publication year, and country; 2) Patient information: age, gender, disease type; 3) Drug information: intervention duration, number of participants, dosage, and outcomes for the intervention group and also the control group. Missing data were addressed by contacting the original study authors. As per the inclusion criteria, literature screening and data extraction were carried out independently by two researchers (Wang J.C., Bi C.H.). Any discrepancies were resolved through discussion with a senior researcher (Wei F.Q.).

### 2.4 Quality assessment concerning included studies

The quality assessment of the included RCTs was carried out using the modified Jadad scale (Moher et al., 2010). Expanding upon the original Jadad scale, this modified version evaluates random sequence generation, allocation concealment, blinding, and withdrawals and loss to follow-ups, providing a comprehensive assessment of study design and execution quality. Scores ranged from 0 to 7 points, with 4–7 indicating high-quality studies and 1–3 indicating low-quality studies. Additionally, we utilized the ROB to appraise the potential biases within the studies (Higgins et al., 2011). Random sequence generation, allocation concealment, blinding of participants and personnel, blinding of outcome assessment, completeness of outcome data, selective reporting, and other potential sources of bias were all assessed via the tool. Each item was categorized as having a high, low, or unclear risk of bias. When details were insufficient to judge the conduct of the study, the risk was typically deemed unclear.

### 2.5 Statistical methods

We conducted qualitative and quantitative analyses of adjusted data using Revman v5.3 (Cochrane Collaboration, Oxford, United Kingdom) and Stata/SE v15.1 (StataCorp, College Station, TX, United States) for meta-analysis. Revman 5.3 was utilized for bias risk assessment, while Stata/SE 15.1 was employed for statistical analyses. The I^2^ test was utilized for heterogeneity assessments across the studies. I^2^ between 0% and 40% suggest unimportant heterogeneity; 30%–60% may represent moderate heterogeneity; 50%–90% may represent substantial heterogeneity and 75%–100% indicate considerable heterogeneity. Therefore, a random-effects model would adopted when I^2^ was exceeded 40%, while a fixed-effects model would be selected if I^2^ was below 40% (Higgins et al., 2003). Continuous outcomes were evaluated using mean difference (MD) or standardized mean difference (SMD), depending on whether the variables were measured with the same method and units (MD) or different methods/units (SMD) (Higgins et al., 2011). Effect sizes were reported with 95% confidence intervals (CIs). To identify potential publication bias among the included studies, funnel plots as well as Egger’s test were employed (Egger et al., 1997). If publication bias was identified, the trim-and-fill method was employed to make corresponding adjustments. Synthesized effects were calculated, with statistical significance defined when a two-sided *p* < 0.05. Exploratory subgroup and sensitivity analyses were undertaken to delve into the origins of heterogeneity and validate the robustness of the findings.

## 3 Results

### 3.1 Literature screening

**FIGURE 1:**
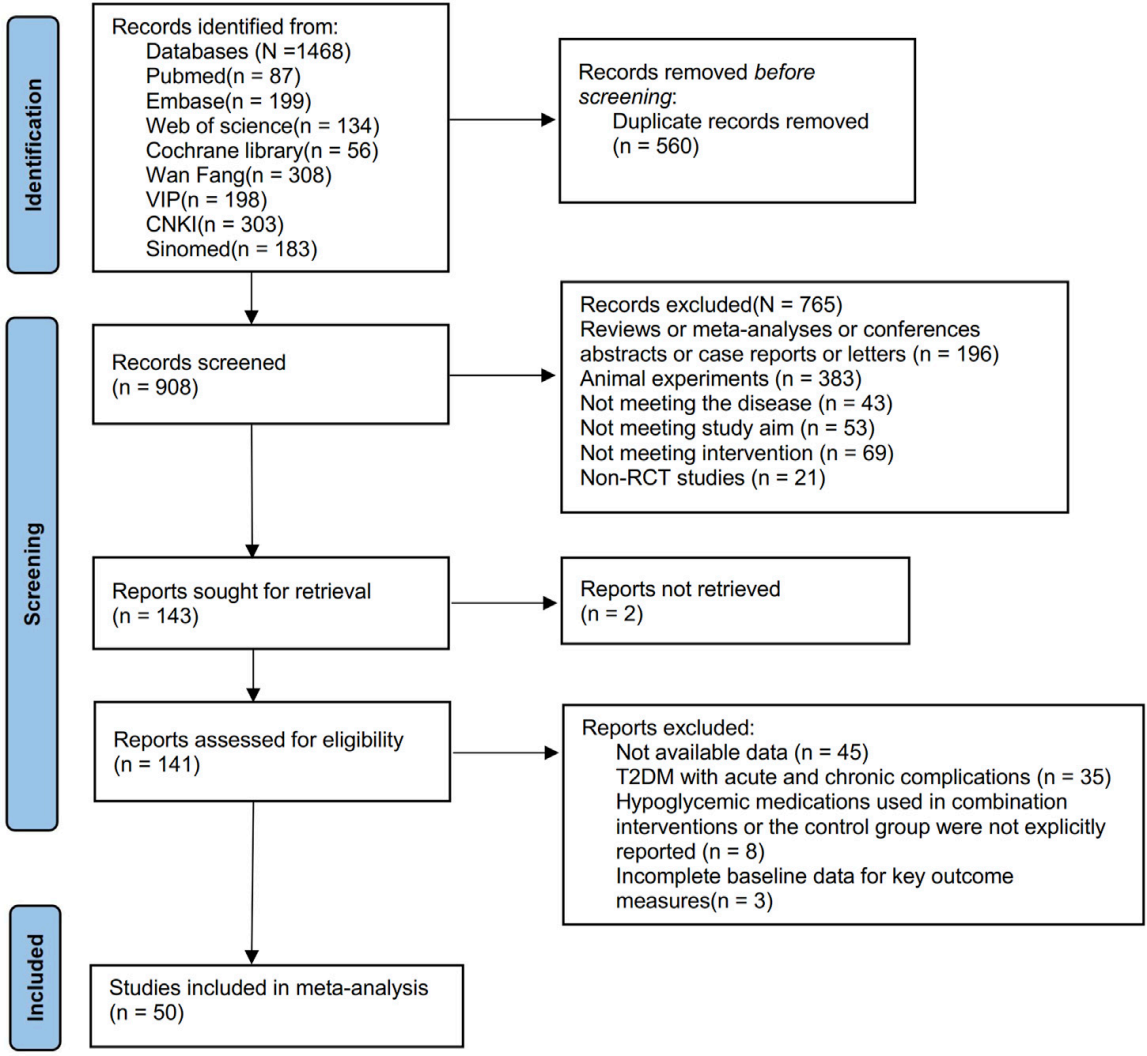
Study identification and selection flowchart.

From the inception to 06/09/2024, searches were done across eight database and search engines: PubMed, Embase, Cochrane Library, Web of Science, China National Knowledge Infrastructure Database (CNKI), Wan-Fang Database, Chinese Scientific Journals Full-text Database (VIP), and the Chinese Biomedical Literature Database (SinoMed), yielding a total of 1,468 articles. After removing 560 duplicates, 908 remained. Screening based on titles and abstracts further excluded 765 articles, resulting in 143 remaining articles. Two articles were inaccessible, leaving 141 articles. Following full-text review, 91 articles were then excluded, resulting in the inclusion of 50 articles (Rong et al., 1997; Zhang et al., 2000; Gao et al., 2002; Liu, 2004; Guo and Zhao, 2006; Li and Liu, 2007; Li et al., 2008; Li, 2008; Yin et al., 2008; Zhu et al., 2009; Sheng and Xie, 2010; Zhang et al., 2010; Meng et al., 2011; Xiang et al., 2011; Yin et al., 2011; Zhang et al., 2011; Liu, 2012; Wang et al., 2012; Xue et al., 2012; Zhang and Yuan, 2012; Zhou and Huang, 2012; Shu, 2014; Yao, 2015; Yu, 2015; Zhu et al., 2015; Du, 2016; Meng, 2016; Sun, 2016; Li, 2017; Sun, 2017; Wu, 2017; Xing, 2017; Zhang, 2017; Fan et al., 2018; Huang et al., 2018; Rashidi et al., 2018; Yao et al., 2018; Jiang and Wang, 2019; Yang et al., 2020; Zhang et al., 2020; Zhu et al., 2020; Chen C. et al., 2021; Chen, 2021). The flowchart of literature screening is plotted in Figure 1.

### 3.2 Characteristics of studies

Basic characteristics of the studies are summarized in Table 1. A total of 50 studies were included, involving 4150 subjects: 2105 in the experimental group and 2045 in the control group. Participant numbers ranged from 31 to 201, with ages ranging from 25 to 75. All studies included subjects with uncomplicated T2DM. Four of the articles were published in English (Yin et al., 2008; Zhang et al., 2010; Rashidi et al., 2018; Zhang et al., 2020), while the remaining studies appeared in Chinese, all of which were RCTs. Among these studies, one was conducted in Iran (Rashidi et al., 2018), whereas the others were conducted in China. Most studies used a two-arm parallel design, ten studies (Rong et al., 1997; Li and Liu, 2007; Li, 2008; Zhu et al., 2009; Zhang et al., 2010; Xiang et al., 2011; Xue et al., 2012; Xing, 2017; Zhang, 2017; Ye, 2021) used a three-arm control design and one study (Zhang et al., 2020) used four-arm designs, where the most suitable paired groups were considered as independent studies, in a bid to reduce selection bias.

**TABLE 1:** The characteristics of the included studies.

Study	Country	Sample (I/C)	Gender (M/F)	Age (years) (I/C)	Patients	Duration of disease (years) (I/C)	Drug and Dosage (I/C)	Lifestyle intervention	Duration	Outcomes
Rong et al. (1997)	China	32/60	Unknown	46 ± 11	NIDDM	5 ± 3	BBR 0.3g, tid/Glb 2.5–10mg, bid	Unknown	10 m	①④
Zhang et al. (2000)	China	78/34	74/38	68.3/69.1	T2DM	6.5/6.2	BBR 700mg, tid/LHI 250mL, qd	Unknown	40d	①⑧⑨
Gao et al. (2002)	China	30/30	39/21	45 ± 11.8 /48 ± 9.7	T2DM	7.4 ± 5.7 /8.1 ± 6.1	BBR 40 mg/kg/d/XKP 8 pills, tid	Yes	30d	①④⑥⑦⑧⑨
Liu (2004)	China	35/33	37/31	53.2 ± 2.8 /55.2 ± 3.6	T2DM	Unknown	BBR 0.5g, tid + Met 0.25–0.5g, tid /Met 0.25–0.5g, tid	Unknown	14d	①②
Guo and Zhao (2006)	China	40/30	40/30	46.8 ± 5.5 /47.2 ± 5.1	NIDDM	Unknown	BBR 0.3–0.5g, tid/Glc 80–160mg, tid	Unknown	60d	①②
Li and Liu (2007)	China	50/51	Unknown	Unknown	T2DM	0.5–4	BBR 0.3g, tid + Glp 15mg, qd /Glp 15mg, qd	Unknown	60d	①②③④⑥⑦⑧⑨
Li et al. (2008)	China	33/32	33/32	47.5/49.2	T2DM	9.01 ± 1.99 /8.11 ± 2.24	BBR 500mg, tid/Met 0.25g, tid	Yes	3 m	①③④⑥⑦⑧⑨
Li (2008)	China	17/18	Unknown	Unknown	T2DM	Unknown	BBR 0.3g, tid + Met 0.5g, tid /Met 0.5g, tid	Yes	12w	①②
Yin et al. (2008)	China	15/16	Unknown	25–75	T2DM	newly diagnosed	BBR 0.5g, tid/Met 0.5g, tid	Unknown	12w	①②③④⑥⑦⑧⑨
Zhu et al. (2009)	China	55/50	Unknown	Unknown	T2DM	5–11	BBR 500mg, tid + Met 50mg, tid + SU \ /Met 50mg, tid + SU \	Yes	3 m	①②③
Sheng and Xie (2010)	China	30/30	29/31	52 ± 11 /51 ± 8	T2DM	5 ± 3 /6 ± 3	BBR 0.5g, tid + Met 0.5g, tid + SU \ /Met 0.5g, tid + SU \	Unknown	3 m	①④⑤⑩⑪⑫
Zhang et al. (2010)	China	50/21	38/33	57 ± 8 /49 ± 10	T2DM	Unknown	BBR 1 g/d/Rog 4 mg/d	Unknown	2 m	①③⑨
Meng et al. (2011)	China	30/30	34/26	51 ± 13.3 /53 ± 13.9	T2DM	newly diagnosed	BBR 0.3g, tid + Ins 6–14 units, tid /Ins 6–14 units, tid	Yes	12w	①②③⑪⑫
Xiang et al. (2011)	China	20/20	Unknown	Unknown	T2DM	newly diagnosed	BBR 0.4g, tid/placebo \	Yes	12w	①②③⑥⑧⑨⑩
Yin et al. (2011)	China	30/30	32/28	36–74	T2DM	newly diagnosed	BBR 0.3g, tid + Met 0.5g, tid /Met 0.5g, tid	Yes	6 m	①②③④⑥⑦⑧⑨
Zhang et al. (2011)	China	30/30	35/25	56.1 ± 0.9 /59.3 ± 1.3	T2DM	5.1 ± 1.3 /6.1 ± 1.2	BBR 0.02 g/kg/d/Rog 4 mg/d	Yes	3 m	①③⑤⑥⑧⑨
Liu (2012)	China	16/16	15/17	66.5 ± 10.1 /66.5 ± 9.9	T2DM	5.5 ± 3.5 /5.5 ± 4.5	BBR 0.5–1g, tid/Glp 10–30mg, bid/tid	Unknown	3 m	①
Wang et al. (2012)	China	22/20	30/12	53.73 ± 8.61 /54.57 ± 9.33	T2DM	newly diagnosed	BBR 0.3–0.5g, tid/Met 0.5g, tid	Unknown	6 m	①②③④⑤ ⑥⑦⑧⑨
Xue et al. (2012)	China	44/45	Unknown	Unknown	T2DM	>1	BBR 0.15g, tid + Met 250mg, tid + SU \ /Met 250mg, tid + SU \	Yes	3 m	①②③
Zhang and Yuan (2012)	China	38/38	41/35	Unknown	T2DM	Unknown	BBR 0.5–0.8g, tid + Met 0.5g, tid /Met 0.5g, tid	Yes	3 m	①②③
Zhou and Huang (2012)	China	46/46	48/44	46.67 ± 8.52	T2DM	4.81 ± 0.29	BBR 0.2g, tid + Met 0.5g, tid /Met 0.5g, tid	Yes	12w	①②⑥⑦⑧⑨⑩
Shu (2014)	China	32/32	39/25	62.80 ± 12.20 /61.21 ± 13.52	T2DM	6.53 ± 2.61 /6.79 ± 2.93	BBR 0.3g, tid + Ins \ /Ins \	Unknown	24w	①④⑩
Yao and Xie (2015)	China	38/38	35/41	44.9 ± 2.8 /43.2 ± 3.2	T2DM	5.58 ± 2.96 /5.37 ± 2.46	BBR 0.5g, tid + Met 0.5g, tid + SU \ /Met 0.5g, tid + SU \	Yes	3 m	①②③④⑥⑦⑧⑨
Yu (2015)	China	49/48	48/49	51.7 ± 4.6 /50.9 ± 3.8	T2DM	4.1 ± 0.5 /4.3 ± 0.7	BBR 0.3g, tid + Met 0.5g, qd /Met 0.5g, qd	Unknown	2 m	①③⑧⑨
Zhu et al. (2015)	China	59/59	68/50	66.4 ± 7.6 /65.6 ± 7.2	T2DM	4.5 ± 1.8 /4.3 ± 2.0	BBR 0.1g, tid + Glc 30mg, qd /Glc 30mg, qd	Yes	12w	①②③⑥⑦⑧⑨
Du (2016)	China	37/36	39/34	67.8 ± 4.6 /66.5 ± 7.1	T2DM	10.9 ± 6.3 /11.6 ± 6.9	BBR 120mg, tid/Met 0.5g, qd	Yes	28d	①②③
Meng (2016)	China	60/60	54/66	65.85 ± 4.78 /67.92 ± 4.73	T2DM	5.32 ± 1.08 /5.28 ± 1.31	BBR 300mg, tid + Glp 5mg, tid /Glp 5mg, tid	Unknown	3 m	③④⑥⑦⑧⑨
Sun (2016)	China	48/48	51/45	52.32 ± 4.45 /52.37 ± 4.48	T2DM	5.68 ± 4.25 /5.71 ± 4.26	BBR 0.3g, tid + Sit 100mg, qd /Sit 100mg, qd	Yes	12w	①②③⑩⑪
Li (2017)	China	30/30	35/25	50.54 ± 3.78 /51.24 ± 3.91	T2DM	6.04 ± 2.43 /6.61 ± 2.75	BBR 0.3g, tid + Sit 100mg, qd /Sit 100mg, qd	Yes	3 m	①②③⑩⑪
Sun (2017)	China	91/91	104/78	58.95 ± 10.57 /58.34 ± 11.21	T2DM	3.98 ± 1.62 /3.64 ± 1.75	BBR 30mg, tid + Met 0.5g, tid /Met 0.5g, tid	Yes	8w	①②③⑤⑥⑦⑧⑨⑩⑪⑫
Wu (2017)	China	33/33	39/27	81.6 ± 6.1 /79.1 ± 3.4	T2DM	Unknown	BBR 0.3g, tid + Glp 5mg, tid /Glp 5mg, tid	Unknown	2 m	④⑨
Xing (2017)	China	35/35	35/35	55.3 ± 6.2 /54.1 ± 6.1	T2DM	5.9 ± 2.8 /5.8 ± 2.7	BBR 0.5g, tid/Met 0.5g, tid	Unknown	12w	①③④⑤⑥⑦⑧⑨
Zhang (2017)	China	40/40	45/35	58.91 ± 6.58 /58.13 ± 6.24	T2DM	6.0 ± 1.7 /5.7 ± 1.8	BBR 3g, tid + Met 750 mg/d + SU \ /Met 750 mg/d + SU \	Yes	3 m	①②③④
Fan et al. (2018)	China	40/40	46/34	53.27 ± 8.15 /52.71 ± 7.89	T2DM	5.59 ± 3.74 /5.64 ± 3.58	BBR 0.5g, tid + Met 0.5g, tid /Met 0.5g, tid	Yes	3 m	①②③⑤⑩⑪⑫
Huang et al. (2018)	China	65/65	63/67	66.09 + 8.6 /67.16∼8.5	T2DM	7.57–6.64 /8.38 ± 7.01	BBR 6g, tid + Ins \/Ins \	Unknown	1 m	①④⑥⑦⑧⑨⑫
Rashidi et al. (2018)	Iran	40/41	33/48	50.18 ± 4.22 /45.12 ± 9.55	T2DM	≤10 years	BBR 0.5g, bid/Placebo \	Yes	4w	①②④⑤⑥⑦⑧⑨
Yao et al. (2018)	China	48/48	49/47	59.10 ± 2.32 /58.24 ± 1.51	T2DM	8.3 ± 2.7 /8.2 ± 2.6	BBR 0.3g, tid + Glp 5mg, tid /Glp 5mg, tid	Unknown	3 m	④⑨
Jiang and Wang (2019)	China	51/51	53/49	63.19 ± 4.82 /62.76 ± 4.59	T2DM	10.03 ± 1.68 /9.84 ± 1.72	BBR 0.3g, tid + Met 0.5g, tid /Met 0.5g, tid	Yes	12w	①②③
Yang et al. (2020)	China	96/96	97/95	49.9 ± 7.8 /49.7 ± 7.4	T2DM	newly diagnosed	BBR 0.5g, tid + Met 0.5–1g, bid /Met 0.5–1g, bid	Yes	3 m	①②③⑥⑧⑪⑫
Zhang et al. (2020)	China	98/103	120/81	51.94 ± 14.30 /53.65 ± 11.28	T2DM	newly diagnosed	BBR 0.6g, bid/BBR 0.6g, bid	Unknown	12w	①②③④⑤⑥⑦⑧⑨
Zhu et al. (2020)	China	25/25	27/23	58.80 ± 12.27 /60.46 ± 11.73	T2DM	newly diagnosed	BBR 0.5g, tid/Placebo \	Yes	12w	①③④⑤
Chen et al. (2021a)	China	62/62	65/59	64.67 ± 4.85 /64.26 ± 5.03	T2DM	6.87 ± 3.15 /6.34 ± 2.96	BBR 0.2–0.5g, tid + Met 0.5g, tid /Met 0.5g, tid	Yes	1 m	①②③
Chen (2021)	China	30/30	35/25	62.5 ± 4.3 /62.2 ± 4.1	T2DM	newly diagnosed	BBR 0.3g, tid + Met 0.5g, tid /Met 0.5g, tid	Unknown	12w	①②③
Chen et al. (2021b)	China	30/30	34/26	58.19 ± 11.03 /57.25 ± 10.10	T2DM	5.62 ± 1.27 /5.64 ± 1.24	BBR 100mg, tid + Glc 60mg, qd /Glc 60mg, qd	Yes	8w	①③⑧⑨
Ye (2021)	China	30/30	28/32	65.97 ± 1.76 /65.39 ± 1.36	T2DM	Unknown	BBR 3g, tid + Met 750 mg/d + SU \ /Met 750 mg/d + SU \	Unknown	180d	①②③④
Wang et al. (2022)	China	56/52	64/44	43.00 ± 8. 35 /41.49 ± 5. 68	T2DM	Unknown	BBR 0.5g, tid + Met 1.5–2 g/d /Met 1.5–2 g/d	Yes	24w	①②③④⑤⑥⑦⑧⑨⑩⑪⑫
Yu (2022)	China	30/30	36/24	44.64 ± 3.36 /42.15 ± 3.85	T2DM	6.81 ± 1.19 /6.41 ± 1.59	BBR 0.3g, tid + Met 0.5g, bid /Met 0.5g, bid	Yes	3 m	①②③⑧⑨⑩⑪⑫
Chen et al. (2023)	China	30/30	34/26	54.30 ± 3.15 /54.31 ± 3.32	T2DM	6.12 ± 1.35 /6.29 ± 1.41	BBR 0.2g, tid/Met 1.5g–2g/d	Unknown	2 m	①②③⑤⑧⑨
Lu (2023)	China	30/30	35/25	58.11 ± 4.06 /57.38 ± 4.25	T2DM	Unknown	BBR 500mg, tid + Ins 15 units/d /Ins 15 units/d	Unknown	Unknown	①②③⑤
Yang et al. (2023b)	China	51/52	Unknown	47.16 ± 10.78 /47.27 ± 9.37	T2DM	Unknown	BBR 0.5g, tid + Met 0.5g, tid /Met 0.5g, tid	Yes	12w	①②③④⑤⑥⑦⑧⑨

I: intervention group; C: control group; M: male; F: female; BBR: berberine; SU: sulfonylureas; Glp: Glipizide; Glc: Gliclazide; Glb: Glyburide; Rog: Rosiglitazone; Sit: Sitagliptin; Met: Metformin; LHI: ligustrazine hydrochloride injection; XKP: xiaoke pill; Ins: Insulin.

Outcomes: ①FPG: fasting plasma glucose; ②2hPBG: 2-h postprandial blood glucose; ③HbA1c: glycated haemoglobin; ④Fins: fasting serum insulin; ⑤HOMA-IR: homeostasis model assessment index for assessing insulin resistance; ⑥LDL-C: low-density lipoprotein cholesterol; ⑦HDL-C: high-density lipoprotein cholesterol; ⑧TC: total cholesterol; ⑨TG: triglyceride; ⑩CRP: C-reactive protein; ⑪1L-6: interleukin-6; ⑫ TNF-α: tumor necrosis factor-alpha.

In 50 studies, 17 reported on the use of BBR alone, including 4 studies (Xiang et al., 2011; Rashidi et al., 2018; Zhang et al., 2020; Zhu et al., 2020) that compared BBR to a placebo and 13 studies that conducted head-to-head trials of BBR with other medications. Among these, 3 studies (Rong et al., 1997; Guo and Zhao, 2006; Liu, 2012) compared BBR to SU (including glyburide, gliclazide, and glipizide), 6 studies (Li et al., 2008; Yin et al., 2008; Wang et al., 2012; Du, 2016; Xing, 2017; Chen et al., 2023) compared to Met, 2 studies (Zhang et al., 2010; Zhang et al., 2011) contrasted with TZDs (rosiglitazone), and 2 studies (Zhang et al., 2000; Gao et al., 2002) compared to traditional Chinese medicine. Additionally, 33 studies compared BBR with one or two conventional medications used in combination, with the same conventional medication serving as the control. This included 15 studies (Liu, 2004; Li, 2008; Yin et al., 2011; Zhang and Yuan, 2012; Zhou and Huang, 2012; Yu, 2015; Sun, 2017; Fan et al., 2018; Jiang and Wang, 2019; Yang et al., 2020; Chen C. et al., 2021; Chen, 2021; Wang et al., 2022; Yu, 2022; Yang B. et al., 2023) combining BBR with Met, 6 studies (Li and Liu, 2007; Zhu et al., 2015; Meng, 2016; Wu, 2017; Yao et al., 2018; Chen Z. et al., 2021) with SU (including gliclazide and glipizide), 2 studies (Sun, 2016; Li, 2017) with DPP-4 (sitagliptin), 4 studies (Meng et al., 2011; Shu, 2014; Huang et al., 2018; Lu, 2023) with insulin, and 6 studies (Zhu et al., 2009; Sheng and Xie, 2010; Xue et al., 2012; Yao, 2015; Zhang, 2017; Ye, 2021) combining Met and SU (including glyburide, gliclazide, and glipizide).

Treatment durations ranged from 14 days to 6 months. One trial (Liu, 2004) lasted 14 days, one trial (Rong et al., 1997) lasted 10 months, five trials (Gao et al., 2002; Du, 2016; Huang et al., 2018; Rashidi et al., 2018; Chen C. et al., 2021) lasted 1 month, five trials (Yin et al., 2011; Wang et al., 2012; Shu, 2014; Ye, 2021; Wang et al., 2022) lasted 180 days, six trials (Zhang et al., 2010; Yu, 2015; Sun, 2017; Wu, 2017; Chen Z. et al., 2021; Chen et al., 2023) lasted 2 months, one trial (Zhang et al., 2000) lasted 40 days and one trial (Lu, 2023) had an unknown duration; the remaining 30 studies lasted 3 months. The most common dosage of BBR was 0.9–1.5 g/d. One study (Sun, 2017) reported 0.09 g/d, while 6 trials (Xue et al., 2012; Zhou and Huang, 2012; Zhu et al., 2015; Du, 2016; Chen Z. et al., 2021; Chen et al., 2023) used 0.3–0.6 g/d and 3 studies (Zhang et al., 2000; Liu, 2012; Zhang and Yuan, 2012) used 1.5–3 g/d. In two studies (Zhang, 2017; Ye, 2021), patients received 9.0 g/d, and one study (Huang et al., 2018) received 18.0 g/d. Two studies dosed by patients’ weight: one (Gao et al., 2002) at 40 mg/kg/d and the other (Zhang et al., 2011) at 0.02 g/kg/d; In the remaining 35 studies, the dosage of BBR ranged 0.9–1.5 g/d. The detailed information was shown in Table 1.

### 3.3 Risk assessment

**FIGURE 2:**
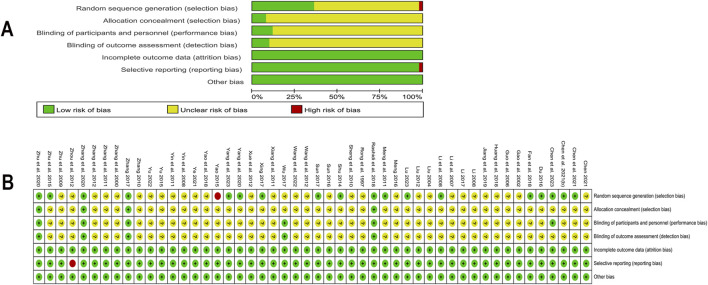
Risk of bias in included studies **(A)** Summary of risk of bias in included studies; **(B)** Detailed assessment of risk of bias in included studies.

The 50 RCTs included in this study ranged in quality from low to high. Four studies (Zhang, 2017; Rashidi et al., 2018; Zhang et al., 2020; Zhu et al., 2020) were assessed as low risk, with detailed implementation of randomization and blinding, and reports on the number and reasons for withdrawals or terminations. 31 studies mentioned “random grouping” but did not detail the random sequence generation or allocation concealment methods, thus assessed as “unclear risk” of selection bias and one (Yao, 2015) used admission time to randomize the number of participants with high risk. 44 studies had insufficient descriptions of blinding for participants, personnel, and outcome assessment, thus assessed as “unclear risk” for performance bias or detection bias. One study (Zhou and Huang, 2012) was rated high risk for not reporting Fins outcomes. Other included studies were evaluated as having a low risk regarding the completeness of outcome data, selective reporting, or other biases. Detailed assessment of the risk of bias was illustrated in Figure 2.

We also evaluated the quality of the studies using modified Jadad scores. Scores varied from three to seven. A total of 31 studies were classified as low quality, with a minimum score of 2 (Yao, 2015). Although 19 other studies were of higher quality, the majority received a score of 4. One study (Chen et al., 2023) achieved a score of 5, two studies (Zhang, 2017; Rashidi et al., 2018) were awarded 6 points, and two studies (Zhang et al., 2020; Zhu et al., 2020) received the full score of 7. Detailed information was shown in Supplementary Table 3.

### 3.4 Meta-analysis

#### 3.4.1 BBR alone vs. conventional treatment or placebo

##### 3.4.1.1 FPG

**FIGURE 3:**
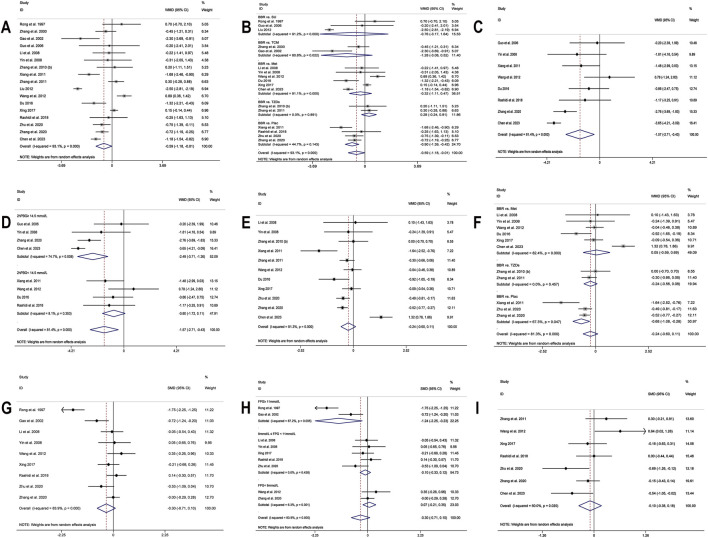
Forest plot for BBR alone vs conventional treatment or placebo: **(A)** FPG; **(B)** subgroup analysis of FPG by comparison regimen; **(C)** 2hPBG; **(D)** subgroup analysis of 2hPBG by baseline levels; **(E)** HbA1c; **(F)** subgroup analysis of HbA1c by comparison regimen; **(G)** Fins; **(H)** subgroup analysis of Fins by baseline levels; **(I)** HOMA-IR.

**FIGURE 4:**
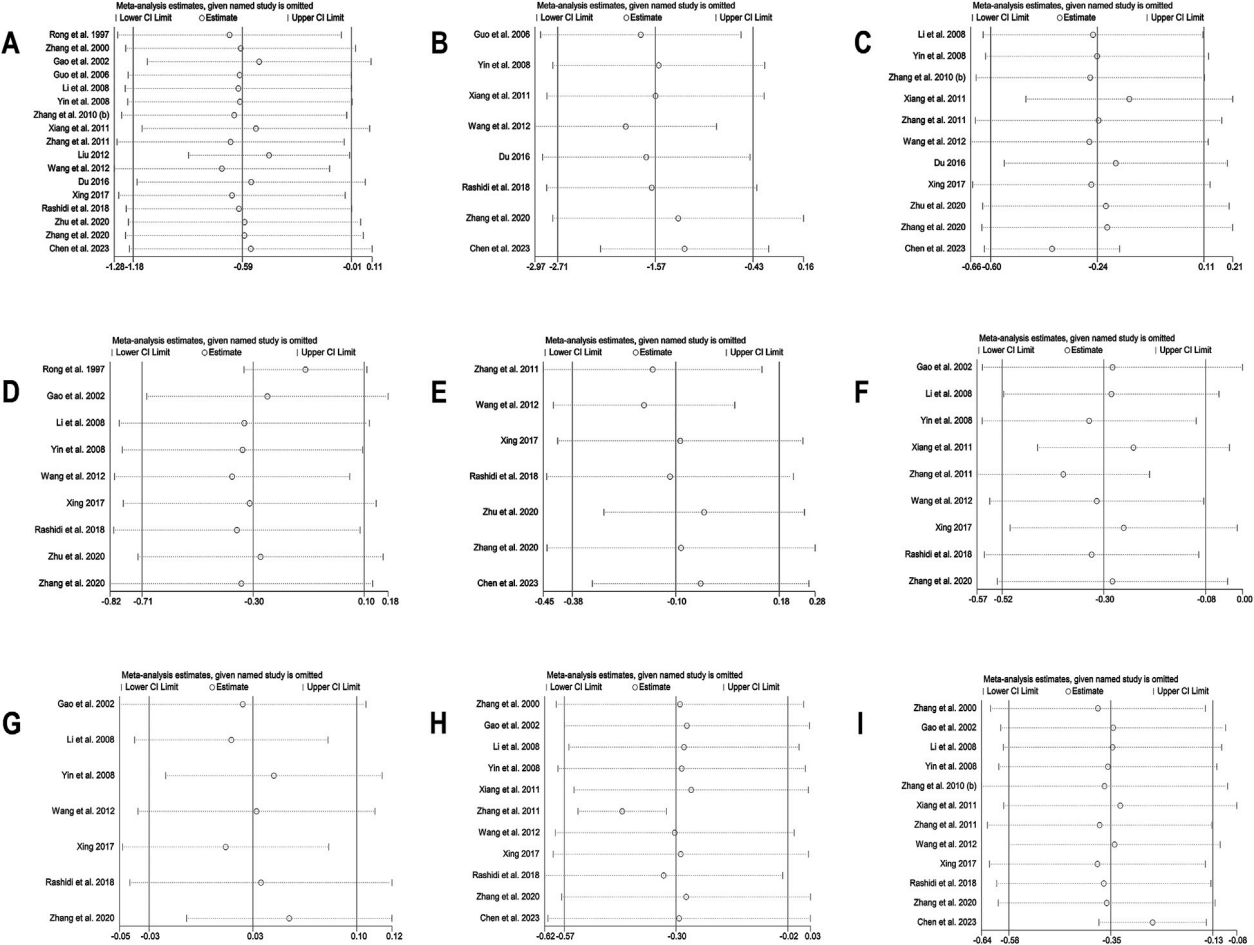
Sensitivity analysis for BBR alone vs conventional treatment or placebo: **(A)** FPG; **(B)** 2hPBG; **(C)** HbA1c; **(D)** Fins; **(E)** HOMA-IR; **(F)** LDL-C; **(G)** HDL-C; **(H)** TC; **(I)** TG.

A total of 17 studies (Rong et al., 1997; Zhang et al., 2000; Gao et al., 2002; Guo and Zhao, 2006; Li et al., 2008; Yin et al., 2008; Zhang et al., 2010; Xiang et al., 2011; Zhang et al., 2011; Liu, 2012; Wang et al., 2012; Du, 2016; Xing, 2017; Rashidi et al., 2018; Zhang et al., 2020; Zhu et al., 2020; Chen et al., 2023) were included. Meta-analysis utilizing a random effects model (I^2^ = 93.1%, *p* = 0.000) demonstrated that, compared to the control group, BBR’s effect on lowering FPG approached statistical significance (MD = −0.59 mmol/L, 95% CI: −1.18, −0.01, *p* = 0.048 < 0.05) (Figure 3A). However, it should be noted that this *p*-value was at the significance threshold, and its clinical relevance should be interpreted with caution. A subgroup analysis based on the control regimen revealed no significant difference in FPG reduction when BBR monotherapy was compared with SU (MD = −0.76 mmol/L, 95% CI (−3.17, 1.64), *p* = 0.533), Met (MD = −0.32 mmol/L, 95% CI (−1.11, 0.47), *p* = 0.434), TZDs (MD = 0.28 mmol/L, 95% CI (−0.24, 0.81), *p* = 0.292), or traditional Chinese medicinal products (MD = −1.28 mmol/L, 95% CI (−3.08, 0.52), *p* = 0.164). However, compared with placebo, the reduction was statistically significant (MD = −0.90 mmol/L, 95% CI (−1.39, −0.42), *p* < 0.01) (Figure 3B). Meta-regression indicated no significant differences in FPG regarding sample size (*p* = 0.188), publication year (*p* = 0.599), intervention duration (*p* = 0.305), or patients’ baseline FPG levels (*p* = 0.054). We hypothesized that the high heterogeneity may be related to the disease course of T2DM, and further high-quality studies are needed for validation. Sensitivity analysis indicated that the results were relatively robust (Figure 4A).

##### 3.4.1.2 2hPBG

Eight studies (Guo and Zhao, 2006; Yin et al., 2008; Xiang et al., 2011; Wang et al., 2012; Du, 2016; Rashidi et al., 2018; Zhang et al., 2020; Chen et al., 2023) reported 2hPBG. Using a random-effects model (I^2^ = 81.4, *p* = 0.000), the results revealed that the experimental group had significantly lower 2hPBG levels than the control group (MD = −1.57 mmol/L, 95% CI (−2.71, −0.43), *p* < 0.01) (Figure 3C). Meta-regression results showed a significant difference in baseline 2hPBG (*p* = 0.033 < 0.05). A subgroup analysis based on patients’ baseline average 2hPBG levels did not completely reduce the heterogeneity within the subgroups (Figure 3D). The results showed a significant difference in improvement in patients with baseline 2hPBG ≥14.5 mmol/L when treated with BBR monotherapy compared to controls (MD = −2.49 mmol/L, 95% CI (−3.71, −1.26), *p* < 0.01); conversely, there was no significant improvement in patients with baseline 2hPBG <14.5 mmol/L (MD = −0.80 mmol/L, 95% CI (−1.72, 0.11), *p* = 0.086). We hypothesized that the high heterogeneity may be related to the disease course of T2DM, and further studies are needed for validation. Sensitivity analysis indicated that the results were relatively robust (Figure 4B).

##### 3.4.1.3 HbA1c

11 studies (Li et al., 2008; Yin et al., 2008; Zhang et al., 2010; Xiang et al., 2011; Zhang et al., 2011; Wang et al., 2012; Du, 2016; Xing, 2017; Zhang et al., 2020; Zhu et al., 2020; Chen et al., 2023), including 749 T2DM patients, were included. Utilizing a random-effects model (I^2^ = 81.3%, *p* = 0.000), The meta-analysis revealed no significant HbA1c level reduction in the experimental group compared to the control group (MD = −0.24%, 95% CI (−0.60, 0.11), *p* = 0.181) (Figure 3E). Subgroup analysis revealed that, compared to placebo, BBR alone significantly improved HbA1c levels (MD = −0.68%, 95% CI (−1.08, −0.28), *p* < 0.01). However, no statistically significant difference was observed in HbA1c reduction when compared with hypoglycemic agents such as Met (MD = 0.05%, 95% CI (−0.59, 0.69), *p* = 0.884) or TZDs (MD = −0.24%, 95% CI (−0.56, 0.08), *p* = 0.143) (Figure 3F). Sensitivity analysis indicated a deviation in the study by Chen et al. (2023) (Figure 4C). After excluding this study, the I^2^ value decreased from 81.3% to 48.5%, and the synthesized results were reversed (MD = −0.39%, 95% CI (−0.62, −0.17), *p* < 0.01), suggesting instability in the outcome. The relatively low BBR dosage in that study (Chen et al., 2023) may have influenced the overall results. Therefore, BBR monotherapy may be more effective than conventional treatment in improving HbA1c, although further high-quality studies are needed to confirm this finding.

##### 3.4.1.4 Fins

A total of nine studies (Rong et al., 1997; Gao et al., 2002; Li et al., 2008; Yin et al., 2008; Wang et al., 2012; Xing, 2017; Rashidi et al., 2018; Zhang et al., 2020; Zhu et al., 2020) were included. Meta-analysis utilizing a random effects model (I^2^ = 83.9%, *p* = 0.000) showed no significant difference in Fins improvement with BBR compared to the control group (SMD = −0.30, 95% CI (−0.71, 0.10), *p* = 0.136) (Figure 3G). Meta-regression showed a significant difference in baseline mean Fins (*p* = 0.001). Further subgroup analysis indicated that heterogeneity within subgroups was not completely reduced (Figure 3H). In addition, we conducted a sensitivity analysis (Figure 4D), which indicated a deviation in the study by Rong et al. (1997). After excluding this study, the I^2^ value decreased from 83.9% to 40.3%. The large difference in sample sizes between the experimental and control groups in this study (Rong et al., 1997) might have contributed to the overall heterogeneity. The synthesized results did not reverse (SMD = −0.12, 95% CI (−0.34, 0.11), *p* = 0.304), suggesting that the result was relatively robust.

##### 3.4.1.5 HOMA-IR

**FIGURE 5:**
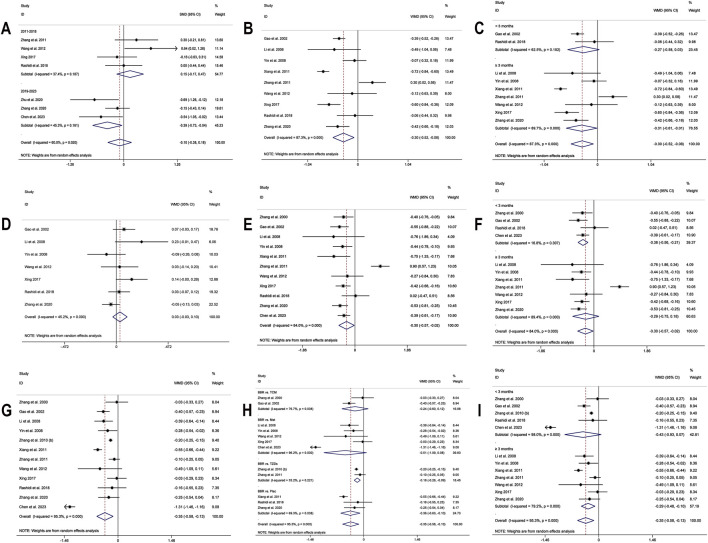
Forest plot for BBR alone vs conventional treatment or placebo: **(A)** subgroup analysis of HOMA-IR by publication year; **(B)** LDL-C; **(C)** subgroup analysis of LDL-C by intervention duration; **(D)** HDL-C; **(E)** TC; **(F)** subgroup analysis of TC by intervention duration; **(G)** TG; **(H)** subgroup analysis of TG by comparison regimen; **(I)** subgroup analysis of TG by intervention duration.

In the meta-analysis, which encompassed seven studies (Zhang et al., 2011; Wang et al., 2012; Xing, 2017; Rashidi et al., 2018; Zhang et al., 2020; Zhu et al., 2020; Chen et al., 2023) and utilized a random effects model (I^2^ = 60.0%, *p* = 0.020), no significant improvement in HOMA-IR was detected with BBR *versus* the control group (SMD = −0.10, 95% CI (−0.38, 0.18), *p* = 0.503) (Figure 3I). Meta-regression results indicated that significant differences in HOMA-IR were observed with respect to the publication year (*p* = 0.023). Further subgroup analysis showed reduced heterogeneity within each subgroup (I^2^ < 50), suggesting that publication year might be a source of heterogeneity (Figure 5A). Sensitivity analysis indicated that the results were robust (Figure 4E).

##### 3.4.1.6 LDL-C

A total of 9 studies (Gao et al., 2002; Li et al., 2008; Yin et al., 2008; Xiang et al., 2011; Zhang et al., 2011; Wang et al., 2012; Xing, 2017; Rashidi et al., 2018; Zhang et al., 2020) reported data on LDL-C. A random-effects model was applied (I^2^ = 87.3%, *p* = 0.000), and the results showed that BBR alone significantly reduced LDL-C levels (MD = −0.30 mmol/L, 95% CI (−0.52, −0.08), *p* < 0.01) (Figure 5B). Subgroup analysis based on intervention duration revealed that BBR monotherapy administered for ≥3 months significantly reduced LDL-C (MD = −0.31 mmol/L, 95% CI (−0.61, −0.01), *p* < 0.05), whereas interventions lasting <3 months did not show a remarkable effect on LDL-C (MD = −0.27 mmol/L, 95% CI (−0.58, 0.03), *p* = 0.082) (Figure 5C). Meta-regression showed no significant differences in LDL-C regarding publication year (*p* = 0.834), sample size (*p* = 0.802), or baseline FPG levels (*p* = 0.832). We hypothesized that the high heterogeneity may be related to the disease course of patients’ T2DM, and further research is needed for validation. Sensitivity analysis demonstrated that the results were stable (Figure 4F).

##### 3.4.1.7 HDL-C

A total of 7 studies (Gao et al., 2002; Li et al., 2008; Yin et al., 2008; Wang et al., 2012; Xing, 2017; Rashidi et al., 2018; Zhang et al., 2020) reported data on HDL-C. Through a random-effects model (I^2^ = 45.2%, *p* = 0.090), the results showed that BBR monotherapy did not significantly improve HDL-C levels compared to the control group (MD = 0.03 mmol/L, 95% CI (−0.03, 0.10), *p* = 0.326) (Figure 5D). Sensitivity analysis indicated a deviation in the study by Zhang et al. (2020), and after excluding this study, the I^2^ value decreased from 45.2% to 23.2%. The re-synthesized results did not reverse (MD = 0.05 mmol/L, 95% CI (0.00, 0.11), *p* = 0.051), suggesting that the result was relatively robust (Figure 4G).

##### 3.4.1.8 TC

A total of 11 studies (Zhang et al., 2000; Gao et al., 2002; Li et al., 2008; Yin et al., 2008; Xiang et al., 2011; Zhang et al., 2011; Wang et al., 2012; Xing, 2017; Rashidi et al., 2018; Zhang et al., 2020; Chen et al., 2023) reported data on TC, and a random-effects model was applied (I^2^ = 84.0%, *p* = 0.000). The results indicated that BBR monotherapy significantly reduced TC levels (MD = −0.30 mmol/L, 95% CI (−0.57, −0.02), *p* = 0.034 < 0.05) (Figure 5E). A subgroup analysis was conducted based on intervention duration (Figure 5F), but heterogeneity within subgroups was not completely reduced. The results showed that BBR monotherapy significantly reduced TC when the intervention duration was <3 months (MD = −0.38 mmol/L, 95% CI (−0.56, −0.21), *p* < 0.01). However, when the intervention duration was ≥3 months, the improvement in TC was not significant (MD = −0.29 mmol/L, 95% CI (−0.75, 0.18), *p* = 0.226). Sensitivity analysis (Figure 4H) indicated a significant deviation in the study by Zhang et al. (2011), and after its exclusion, the I^2^ value decreased from 84% to 0%. This study had a relatively narrow confidence interval and small standard deviation, which may have significantly influenced overall heterogeneity. A fixed-effects model was used to re-synthesis the effect size, and the results did not reverse (MD = −0.43 mmol/L, 95% CI (−0.54, −0.32), *p* < 0.01), suggesting that the result was relatively robust.

##### 3.4.1.9 TG

A total of 12 studies (Zhang et al., 2000; Gao et al., 2002; Li et al., 2008; Yin et al., 2008; Zhang et al., 2010; Xiang et al., 2011; Zhang et al., 2011; Wang et al., 2012; Xing, 2017; Rashidi et al., 2018; Zhang et al., 2020; Chen et al., 2023) reported data on TG. A random-effects model analysis was performed (I^2^ = 95.3%, *p* = 0.000), showing that BBR monotherapy significantly improved TG levels (MD = −0.35 mmol/L, 95% CI (−0.58, −0.13), *p* < 0.01) (Figure 5G). A subgroup analysis based on the comparison regimen (Figure 5H) showed that compared to monotherapy with TZDs (MD = −0.18 mmol/L, 95% CI (−0.26, −0.09), *p* < 0.01) or placebo (MD = −0.36 mmol/L, 95% CI (−0.63, −0.10), *p* < 0.01), BBR demonstrated a statistically significant difference in improving TG levels. However, no significant differences in TG reduction were found when comparing BBR to traditional Chinese medicinal products (MD = −0.24 mmol/L, 95% CI (−0.60, 0.12), *p* = 0.196) or Met (MD = −0.51 mmol/L, 95% CI (−1.09, 0.08), *p* = 0.089). In addition, subgroup analysis based on intervention duration revealed that BBR monotherapy for ≥3 months significantly reduced TG levels (MD = −0.29 mmol/L, 95% CI (−0.48, −0.10), *p* < 0.01), while interventions lasting <3 months did not show remarkable improvement in TG (MD = −0.43 mmol/L, 95% CI (−0.93, 0.07), *p* = 0.091) (Figure 5I). Meta-regression showed no significant differences in TG levels concerning baseline FPG (*p* = 0.908), publication year (*p* = 0.460), or sample size (*p* = 0.075). We hypothesized that the high heterogeneity may be related to the disease course of T2DM, and further studies are needed for validation. Sensitivity analysis indicated a deviation in the study by Chen et al. (2023), and after its exclusion, the re-synthesized results did not reverse (MD = −0.26 mmol/L, 95% CI (−0.38, −0.15), *p* < 0.01), suggesting that the result was relatively robust (Figure 4I).

##### 3.4.1.10 Inflammatory markers

Only one study (Xiang et al., 2011) reported the effects of BBR monotherapy *versus* placebo on inflammatory markers. The results showed that compared to the control, BBR significantly improved levels of CRP, IL-6, and TNF-α (*p* < 0.05).

#### 3.4.2 BBR combined with conventional treatment vs conventional treatment

##### 3.4.2.1 FPG

**FIGURE 6:**
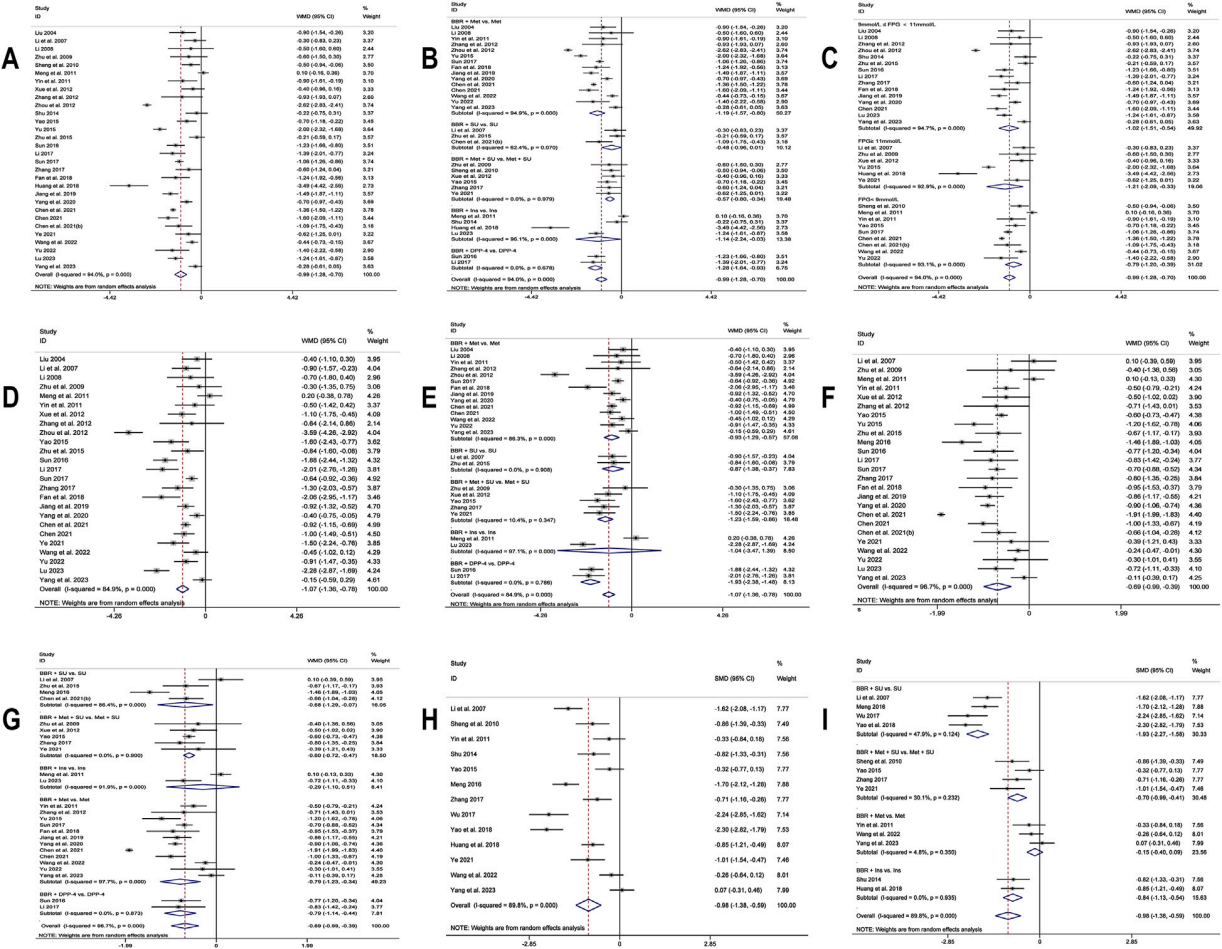
Forest plot for BBR combined with conventional treatment vs conventional treatment: **(A)** FPG; **(B)** subgroup analysis of FPG by comparison regimen; **(C)** subgroup analysis of FPG by baseline levels; **(D)** 2hPBG; **(E)** subgroup analysis of 2hPBG by comparison regimen; **(F)** HbA1c; **(G)** subgroup analysis of HbA1c by comparison regimen; **(H)** Fins; **(I)** subgroup analysis of Fins by comparison regimen.

30 studies (Liu, 2004; Li and Liu, 2007; Li, 2008; Zhu et al., 2009; Sheng and Xie, 2010; Meng et al., 2011; Yin et al., 2011; Xue et al., 2012; Zhang and Yuan, 2012; Zhou and Huang, 2012; Shu, 2014; Yao, 2015; Yu, 2015; Zhu et al., 2015; Sun, 2016; Li, 2017; Sun, 2017; Zhang, 2017; Fan et al., 2018; Huang et al., 2018; Jiang and Wang, 2019; Yang et al., 2020; Chen C. et al., 2021; Chen, 2021; Chen Z. et al., 2021; Ye, 2021; Wang et al., 2022; Yu, 2022; Lu, 2023; Yang B. et al., 2023) were included. Employing a random-effects model as per heterogeneity test results (I^2^ = 94.0%, *p* = 0.000), the meta-analysis revealed that the experimental group had remarkably lower FPG levels than the control group (MD = −0.99 mmol/L, 95% CI (−1.28, −0.70), *p* < 0.01) (Figure 6A). Due to high heterogeneity, subgroup analysis was undertaken regarding interventions and mean baseline FPG levels (Figures 6B,C). However, heterogeneity within subgroups was not completely reduced. The results showed that BBR as an adjunct to DPP-4 inhibitors (MD = −1.28 mmol/L, 95% CI (−1.64, −0.93), *p* < 0.01), insulin (MD = −1.14 mmol/L, 95% CI (−2.24, −0.03), *p* = 0.044 < 0.05), Met (MD = −1.19 mmol/L, 95% CI (−1.57, −0.80), *p* < 0.01), and Met + SU (MD = −0.57 mmol/L, 95% CI (−0.80, −0.34), *p* < 0.01) demonstrated a significant reduction in FPG compared to the use of these medications alone. In contrast, BBR + SU did not show a statistically significant reduction in FPG (MD = −0.48 mmol/L, 95% CI (−0.96, 0.01), *p* = 0.053). Additionally, BBR significantly reduced FPG levels in patients with different baseline FPG levels: <9 mmol/L (MD = −0.79, 95% CI (−1.20, −0.39), *p* < 0.01), between 9 and 11 mmol/L (MD = −1.02, 95% CI (−1.51, −0.54), *p* < 0.01), and ≥11 mmol/L (MD = −1.21, 95% CI (−2.09, −0.33), *p* < 0.01), all showing statistical significance. A meta-regression was then conducted and found no significant differences in FPG reduction based on sample size (*p* = 0.503), publication year (*p* = 0.491), or BBR dosage (*p* = 0.615). We hypothesized that the high heterogeneity may be associated with the dosage of the concomitant medications, and further research is needed for validation. According to the sensitivity analysis results, after sequentially excluding Zhou et al., 2012 (Zhou and Huang, 2012) (MD = −0.92 mmol/L, 95% CI (−1.15, −0.69), *p* < 0.01) and Huang et al., 2018 (Huang et al., 2018) (MD = −0.92 mmol/L, 95% CI (−1.20, −0.63), *p* < 0.01), the synthesized results did not reverse, suggesting that the result was relatively robust (Figure 8A).

##### 3.4.2.2 2hPBG

25 studies (Liu, 2004; Li and Liu, 2007; Li, 2008; Zhu et al., 2009; Meng et al., 2011; Yin et al., 2011; Xue et al., 2012; Zhang and Yuan, 2012; Zhou and Huang, 2012; Yao, 2015; Zhu et al., 2015; Sun, 2016; Li, 2017; Sun, 2017; Zhang, 2017; Fan et al., 2018; Jiang and Wang, 2019; Yang et al., 2020; Chen C. et al., 2021; Chen, 2021; Ye, 2021; Wang et al., 2022; Yu, 2022; Lu, 2023; Yang B. et al., 2023) reported 2hPBG. Through a random-effects model (I^2^ = 84.9, *p* = 0.000), the results demonstrate that the co-administration of BBR significantly reduces 2hPBG (MD = −1.07 mmol/L, 95% CI (−1.36, −0.78), *p* < 0.01) (Figure 6D). We conducted a subgroup analysis based on different combination medication regimens. The results showed that BBR, when combined with DPP-4 inhibitors (MD = −1.93 mmol/L, 95% CI (−2.38, −1.48), *p* < 0.01), SU (MD = −0.87 mmol/L, 95% CI (−1.38, −0.37), *p* < 0.01), Met (MD = −0.93 mmol/L, 95% CI (−1.29, −0.57), *p* < 0.01), or Met + SU (MD = −1.23 mmol/L, 95% CI (−1.59, −0.86), *p* < 0.01), significantly reduced 2hPBG compared to monotherapy. In contrast, the combination of BBR and insulin did not show a statistically significant improvement in 2hPBG (MD = −1.04, 95% CI (−3.47, 1.39), *p* = 0.402) (Figure 6E). Given that heterogeneity within subgroups was not completely reduced, we performed a meta-regression, which showed no significant differences in sample size (*p* = 0.178), publication year (*p* = 0.979), BBR dosage (*p* = 0.653), baseline mean FPG (*p* = 0.351), or baseline mean 2hPBG (*p* = 0.065). We hypothesized that the high heterogeneity may be related to the dosage of the co-medication, and further high-quality studies are needed for validation. Sensitivity analysis indicated a deviation in the study by Zhou et al., 2012 (Zhou and Huang, 2012). After excluding this study, the re-synthesized results did not reverse (MD = −0.95 mmol/L, 95% CI (−1.19, −0.72), *p* < 0.01), suggesting that the result was relatively robust (Figure 8B).

##### 3.4.2.3 HbA1c

25 studies (Li and Liu, 2007; Zhu et al., 2009; Meng et al., 2011; Yin et al., 2011; Xue et al., 2012; Zhang and Yuan, 2012; Yao, 2015; Yu, 2015; Zhu et al., 2015; Meng, 2016; Sun, 2016; Li, 2017; Sun, 2017; Zhang, 2017; Fan et al., 2018; Jiang and Wang, 2019; Yang et al., 2020; Chen C. et al., 2021; Chen, 2021; Chen Z. et al., 2021; Ye, 2021; Wang et al., 2022; Yu, 2022; Lu, 2023; Yang B. et al., 2023) reported HbA1c. By a random-effects model (I^2^ = 96.7%, *p* = 0.000), the meta-analysis revealed that the combination of BBR with hypoglycemic drugs significantly reduced HbA1c (MD = −0.69%, 95% CI (−0.99, −0.39), *p* < 0.01) (Figure 6F). To further explore the relationship between Co-administration of BBR and hypoglycemic drugs and improvement in HbA1c, a subgroup analysis was carried out (Figure 6G), which showed that the co-administration of BBR with DPP-4 (MD = −0.79%, 95% CI (−1.14, −0.44), *p* < 0.01), SU (MD = −0.68%, 95% CI (−1.29, −0.07), *p* = 0.028 < 0.05), Met (MD = −0.79%, 95% CI (−1.23, −0.34), *p* < 0.01) and Met + SU (MD = −0.60%, 95% CI (−0.72, −0.47), *p* < 0.01) demonstrated superior efficacy compared to the use of these medications alone. However, the combination with insulin showed no statistically significant difference in improving HbA1c levels (MD = −0.29%, 95% CI (−1.10, 0.51), *p* = 0.474). The meta-regression showed no significant differences regarding BBR dosage (*p* = 0.225), publication year (*p* = 0.145), and sample size (*p* = 0.391). We hypothesized that the high heterogeneity may be related to different measurement times for HbA1c, and further high-quality studies are needed for validation. Sensitivity analysis indicated that the results were relatively robust (Figure 8C).

##### 3.4.2.4 Fins

**FIGURE 7:**
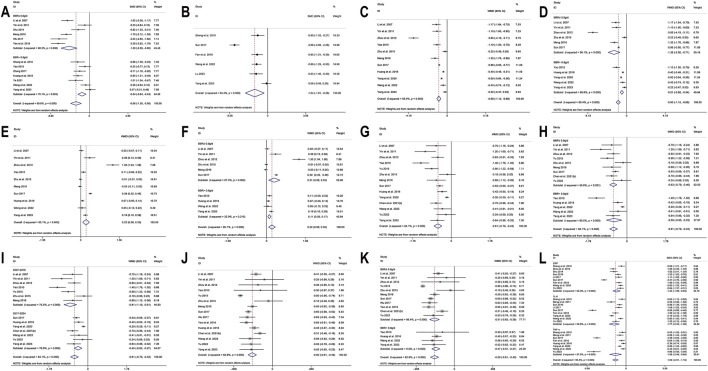
Forest plot for BBR combined with conventional treatment vs conventional treatment: **(A)** subgroup analysis of Fins by BBR dosage; **(B)** HOMA-IR; **(C)** LDL-C; **(D)** subgroup analysis of LDL-C by BBR dosage; **(E)** HDL-C; **(F)** subgroup analysis of HDL-C by BBR dosage; **(G)**TC; **(H)** subgroup analysis of TC by BBR dosage; **(I)** subgroup analysis of TC by publication year; **(J)** TG; **(K)** subgroup analysis of TG by BBR dosage; **(L)** Inflammatory markers.

**FIGURE 8:**
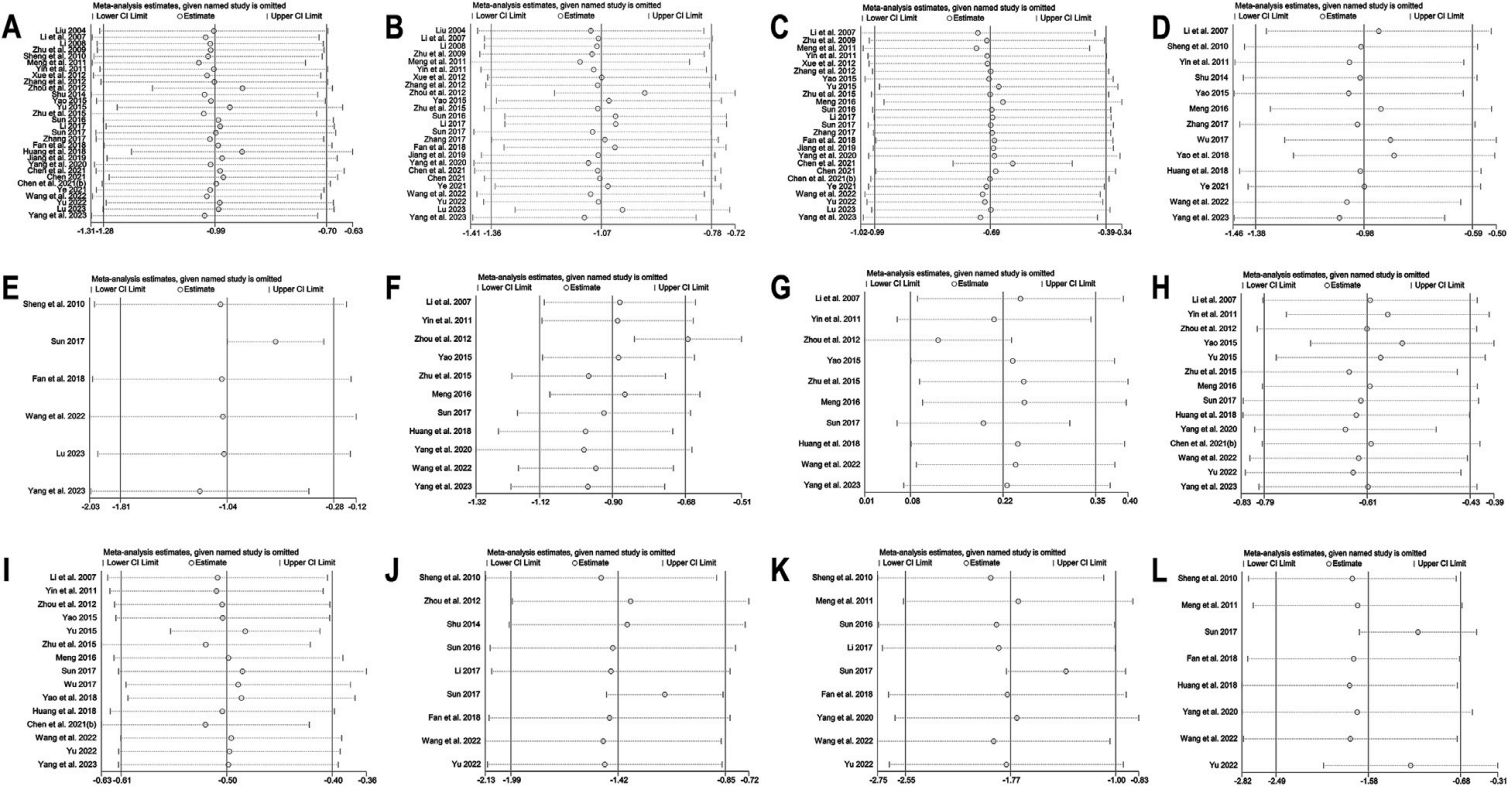
Sensitivity analysis for BBR combined with conventional treatment vs conventional treatment: **(A)** FPG; **(B)** 2hPBG; **(C)** HbA1c; **(D)** Fins; **(E)** HOMA-IR; **(F)** LDL-C; **(G)** HDL-C; **(H)** TC; **(I)** TG; **(J)** CRP; **(K)** 1L-6; **(L)** TNF-α.

13 studies (Li and Liu, 2007; Sheng and Xie, 2010; Yin et al., 2011; Shu, 2014; Yao, 2015; Meng, 2016; Wu, 2017; Zhang, 2017; Huang et al., 2018; Yao et al., 2018; Ye, 2021; Wang et al., 2022; Yang B. et al., 2023) reported Fins. With a random-effects model (I^2^ = 89.8%, *p* = 0.000), the results indicated that BBR effectively decreased Fins levels in T2DM patients (SMD = −0.98, 95% CI (−1.38, −0.59), *p* < 0.01) (Figure 6H). We performed a subgroup analysis based on different co-medication regimens, and the heterogeneity within each subgroup decreased (I^2^ < 50%), suggesting that the combination regimen itself may be a source of heterogeneity (Figure 6I). Results showed that adding BBR to SU (SMD = −1.93, 95% CI (−2.27, −1.58), *p* < 0.01), Met + SU (SMD = −0.70, 95% CI (−0.99, −0.41), *p* < 0.01), or insulin therapy (SMD = −0.84, 95% CI (−1.13, −0.54), *p* < 0.01) significantly reduced Fins levels compared to the use of these drugs alone. In contrast, BBR + Met (SMD = −0.15, 95% CI (−0.40, 0.09), *p* = 0.227) did not show a significant reduction in Fins. A subgroup analysis based on the dosage of BBR (Figure 7A) revealed that both BBR ≤0.9 g/day (SMD = −1.50, 95% CI (−2.09, −0.90), *p* < 0.01) and BBR >0.9 g/day (SMD = −0.54, 95% CI (−0.84, −0.24), *p* < 0.01) significantly reduced Fins levels. Sensitivity analysis indicated that the result was robust (Figure 8D).

##### 3.4.2.5 HOMA-IR

Six studies (Sheng and Xie, 2010; Sun, 2017; Fan et al., 2018; Wang et al., 2022; Lu, 2023; Yang B. et al., 2023) reported HOMA-IR. With a random-effects model (I^2^ = 94.4, *p* = 0.000), the results indicated that BBR effectively decreased HOMA-IR levels in T2DM patients (SMD = −1.04, 95% CI (−1.81, −0.28), *p* < 0.01) (Figure 7B). The meta-regression found no significant differences in HOMA-IR regarding publication year (*p* = 0.226), sample size (*p* = 0.644), or interventions (*p* = 0.925). We hypothesized that the high heterogeneity may be related to the pancreatic function level of patients, and further high-quality studies are needed for validation. Sensitivity analysis indicated a significant deviation in the study by Sun (2017). After excluding this study, the re-synthesized results did not reverse (SMD = −0.70, 95% CI (−1.04, −0.35), *p* < 0.01), suggesting that the result was relatively robust (Figure 8E).

##### 3.4.2.6 LDL-C

A total of 11 studies (Li and Liu, 2007; Yin et al., 2011; Zhou and Huang, 2012; Yao, 2015; Zhu et al., 2015; Meng, 2016; Sun, 2017; Huang et al., 2018; Yang et al., 2020; Wang et al., 2022; Yang B. et al., 2023) reported data on LDL-C. Through a random-effects model (I^2^ = 95.4%, *p* = 0.000) for analysis, the results showed that BBR combined with hypoglycemic agents significantly reduced LDL-C levels (MD = −0.90 mmol/L, 95% CI (−1.12, −0.68), *p* < 0.01) (Figure 7C). A subgroup analysis based on BBR dosage (Figure 7D) revealed that BBR ≤0.9 g/day (MD = −1.36 mmol/L, 95% CI (−2.00, −0.71), *p* < 0.01) had a better effect on LDL-C improvement compared to BBR >0.9 g/day (MD = −0.51 mmol/L, 95% CI (−0.68, −0.34), *p* < 0.01). The meta-regression showed no significant differences in LDL-C based on intervention duration (*p* = 0.960), publication year (*p* = 0.076), sample size (*p* = 0.301), or interventions (*p* = 0.766). We hypothesized that the high heterogeneity may be related to the dosage of the combined medications, and further high-quality studies are needed for validation. Sensitivity analysis identified a significant deviation in the study by Zhou and Huang (2012). After excluding this study, the re-synthesized results did not reverse (MD = −0.67 mmol/L, 95% CI (−0.83, −0.51), *p* < 0.01), indicating that the result was relatively robust (Figure 8F).

##### 3.4.2.7 HDL-C

A total of 10 studies (Li and Liu, 2007; Yin et al., 2011; Zhou and Huang, 2012; Yao, 2015; Zhu et al., 2015; Meng, 2016; Sun, 2017; Huang et al., 2018; Wang et al., 2022; Yang B. et al., 2023) reported data on HDL-C. Through a random-effects model for analysis (I^2^ = 95.1%, *p* = 0.000), the results showed that BBR combination therapy significantly increased HDL-C levels (MD = 0.22 mmol/L, 95% CI (0.08, 0.35), *p* < 0.01) (Figure 7E). A subgroup analysis based on BBR dosage (Figure 7F) indicated that daily BBR ≤0.9 g (MD = 0.31 mmol/L, 95% CI (0.08, 0.53), *p* < 0.01) had a better effect on improving HDL-C compared to BBR >0.9 g/day (MD = 0.11 mmol/L, 95% CI (0.05, 0.17), *p* < 0.01). A meta-regression was conducted and found no significant differences in HDL-C regarding the interventions (*p* = 0.807), publication year (*p* = 0.405), or intervention duration (*p* = 0.851). We hypothesized that the high heterogeneity may be related to the dosage of the combined medications, and further high-quality studies are needed for validation. Sensitivity analysis identified a significant deviation in the study by Zhou and Huang (2012). After excluding this study, the synthesized results did not reverse (MD = 0.12 mmol/L, 95% CI (0.01, 0.23), *p* < 0.05), indicating that the result was relatively robust (Figure 8G).

##### 3.4.2.8 TC

A total of 14 studies (Li and Liu, 2007; Yin et al., 2011; Zhou and Huang, 2012; Yao, 2015; Yu, 2015; Zhu et al., 2015; Meng, 2016; Sun, 2017; Huang et al., 2018; Yang et al., 2020; Chen Z. et al., 2021; Wang et al., 2022; Yu, 2022; Yang B. et al., 2023) reported data on TC. Through a random-effects model (I^2^ = 84.1%, *p* = 0.000), the results showed that combination therapy with BBR significantly reduced TC (MD = −0.61 mmol/L, 95% CI (−0.79, −0.43), *p* < 0.01) (Figure 7G). Subgroup analysis based on BBR dosage indicated that both BBR ≤0.9 g/day (MD = −0.63 mmol/L, 95% CI (−0.79, −0.46), *p* < 0.01) and BBR >0.9 g/day (MD = −0.59 mmol/L, 95% CI (−0.95, −0.23), *p* < 0.01) significantly reduced TC (Figure 7H). Meta-regression results showed a significant difference in TC with respect to the publication year (*p* = 0.026), and further subgroup analysis revealed that heterogeneity within subgroups was not completely reduced (Figure 7I). We hypothesized that the heterogeneity may be related to the dosage of the combined medications, and further high-quality studies are needed for validation. Sensitivity analysis indicated that the results were relatively robust (Figure 8H).

##### 3.4.2.9 TG

A total of 15 studies (Li and Liu, 2007; Yin et al., 2011; Zhou and Huang, 2012; Yao, 2015; Yu, 2015; Zhu et al., 2015; Meng, 2016; Sun, 2017; Wu, 2017; Huang et al., 2018; Yao et al., 2018; Chen Z. et al., 2021; Wang et al., 2022; Yu, 2022; Yang B. et al., 2023) reported data on TG. Through a random-effects model (I^2^ = 82.9%, *p* = 0.000), the results showed that BBR in combination with other medications significantly reduced TG (MD = −0.50 mmol/L, 95% CI (−0.61, −0.40), *p* < 0.01) (Figure 7J). A subgroup analysis based on BBR dosage indicated that both BBR ≤0.9 g/day (MD = −0.51 mmol/L, 95% CI (−0.63, −0.38), *p* < 0.01) and BBR >0.9 g/day (MD = −0.47 mmol/L, 95% CI (−0.57, −0.37), *p* < 0.01) significantly reduced TG levels (Figure 7K). The meta-regression showed no significant differences in TG reduction concerning the type of intervention (*p* = 0.834), intervention duration (*p* = 0.456), publication year (*p* = 0.579), or sample size (*p* = 0.392). We hypothesized that the heterogeneity may be related to the dosage of the combined medications, and further high-quality studies are needed for validation. Sensitivity analysis indicated that the results were relatively robust (Figure 8I).

##### 3.4.2.10 Inflammatory markers

A total of 11 (Sheng and Xie, 2010; Meng et al., 2011; Zhou and Huang, 2012; Shu, 2014; Sun, 2016; Li, 2017; Sun, 2017; Fan et al., 2018; Yang et al., 2020; Wang et al., 2022; Yu, 2022) studies reported on CRP and IL-6, and 8 studies (Sheng and Xie, 2010; Meng et al., 2011; Sun, 2017; Fan et al., 2018; Huang et al., 2018; Yang et al., 2020; Wang et al., 2022; Yu, 2022) reported on TNF-α. We conducted a comprehensive evaluation of inflammatory markers using a random-effects model (I^2^ = 95.4%, *p* = 0.000) for the meta-analysis. The results showed that, compared to hypoglycemic agents alone, the addition of BBR significantly improved inflammatory markers (SMD = −1.59, 95% CI (−2.01, −1.16), *p* < 0.01) (Figure 7L). Specifically, BBR combination therapy significantly improved CRP (SMD = −1.42, 95% CI (−1.99, −0.85), *p* < 0.01), IL-6 (SMD = −1.77, 95% CI (−2.55, −1.00), *p* < 0.01), and TNF-α (SMD = −1.58, 95% CI (−2.49, −0.68), *p* < 0.01). Sensitivity analyses were performed separately, excluding Sun 2017 (Sun, 2017) for both CRP and IL-6, and re-synthesized the results. The results for CRP (SMD = −1.17, 95% CI (−1.48, −0.86), *p* < 0.01) and IL-6 (SMD = −1.36, 95% CI (−1.80, −0.92), *p* < 0.01) did not reverse, indicating that the results were relatively robust (Figures 8J,K,L).

### 3.5 Adverse events

A total of 16 studies reported detailed information on adverse events. The study by Du (2016) noted that the incidence of adverse events with BBR was significantly lower than that with metformin. Additionally, Jiang and Wang (2019) and Chen (2021) reported that the incidence of adverse reactions with BBR combined with hypoglycemic agents was significantly lower than that in the hypoglycemic agents alone group. The summary of adverse events can be found in Supplementary Table 2. No serious adverse events were reported during the treatment period in any of the studies, indicating that BBR is relatively safe.

### 3.6 Publication bias

For metrics with the number of studies ≥10, Egger’s test was utilized to assess the publication bias. For metrics with fewer than 10 studies, funnel plots were employed for the same purpose. Egger’s test revealed significant differences for HbA1c (*p* = 0.007), TC (*p* = 0.005), and TG (*p* = 0.016) (*p* < 0.05), suggesting potential publication bias. After performing the trim-and-fill method, the results did not reverse, indicating that the bias had little effect on the overall results, and the outcomes were relatively robust. In the funnel plot, we observed that the distributions of 2hPBG, Fins, HOMA-IR, and TNF-α were not completely symmetrical, and we conducted trim-and-fill analyses sequentially. The results showed that the meta-analyses for 2hPBG, HOMA-IR, and TNF-α did not undergo significant changes after trimming and filling, suggesting that the risk of bias was minimal for these markers. However, the effect for Fins changed significantly from not statistically significant to statistically significant, indicating that the meta-analysis result for Fins might be affected by publication bias. Therefore, BBR monotherapy may be superior to the control group in improving Fins, and further high-quality studies are needed for validation. We speculated that the source of bias might be related to the limited number of included studies and small sample sizes, which may have led to an overestimation of the effect sizes. Therefore, we recommend that future research include a broader range of data sources to improve the robustness of the meta-analysis and reduce the risk of bias. Other outcomes showed a relatively low risk of bias and had a certain level of reliability. Detailed data on publication bias are provided in Supplementary Data Sheet 1.

## 4 Discussion

The present meta-analysis systematically assessed both the efficacy and safety of BBR used alone or in combination for the treatment of T2DM. Our findings indicate that BBR holds significant potential in the management of T2DM, particularly when used in conjunction with existing hypoglycemic agents.

The meta-analysis results by Lan et al. (2015), Liang et al. (2019), Guo et al. (2021) indicated that BBR monotherapy significantly outperformed placebo in reducing FPG and HbA1c, but its efficacy may be comparable to that of oral hypoglycemic agents. This finding aligns with our subgroup analysis results. Additionally, we found that BBR monotherapy has a certain lipid-regulating effect, which significantly lowered LDL-C, TC, and TG levels, while its effect on HDL-C was not significant. A meta-analysis by Ye et al. (2021) suggested that BBR monotherapy also has a significant effect on improving HDL-C. Through *in silico* network pharmacology analysis, BBR was found to regulate 31 targets related to T2DM and 18 biological pathways associated with the condition. Among these, the PPAR pathway plays a crucial role in BBR’s enhancement of lipid metabolism in T2DM patients (Di et al., 2021). Further, Wu YY proposed that BBR primarily alleviates endoplasmic reticulum (ER) stress by upregulating PPAR-α expression, reducing lipid accumulation, inhibiting cell apoptosis, and thereby promoting lipid oxidative metabolism while reducing lipotoxicity (Wu et al., 2019). While these findings have potential clinical significance, we believe that further high-quality head-to-head clinical trials are necessary to confirm the effects of BBR monotherapy considering the limited number and quality of studies included in the analysis.

Similar to previous research (Guo et al., 2021), our findings showed that, compared to the use of hypoglycemic agents alone, adjunctive BBR significantly improved glucose and lipid metabolism and enhanced insulin sensitivity, which may be attributed to the synergistic effects introduced by BBR (Tian et al., 2019). Studies have shown that BBR primarily improves glycemic control and reduces insulin resistance through various mechanisms, such as activating AMPK, upregulating insulin receptor (InsR) expression, regulating gut microbiota, and promoting GLP-1 secretion (Kong et al., 2009; He et al., 2022; Utami et al., 2023). A recent meta-analysis (Xie et al., 2022) assessing the impact of BBR on glucose metabolism in T2DM, published in 2022, conducted a subgroup analysis based on patients’ baseline FPG levels. The results demonstrated that post-treatment, the experimental group exhibited lower levels of FPG, 2hPBG, and HbA1c compared to the control group (*p* < 0.01). Our study found similar effects of BBR in reducing FPG levels. The distinction in our study lies in finding that the efficacy of BBR in reducing FPG increases with higher baseline FPG levels in patients. This may be linked to its mechanism of glucose concentration-dependent insulin secretion. BBR has been demonstrated to interact directly with the KCNH6 potassium channel, thereby promoting insulin secretion in a hyperglycemia-dependent manner while also contributing to a reduction in the incidence of hypoglycemic events, highlighting its potential application in the adjunctive treatment of diabetes (Zhao et al., 2021).

The meta-analysis by Liang et al. (2019) demonstrated that adding BBR to hypoglycemic agents produced the same improvement in HbA1c as using hypoglycemic agents alone, whereas the meta-analysis by Guo et al. (2021) indicated that the combination of BBR and hypoglycemic agents led to a significantly greater improvement in HbA1c than hypoglycemic agents alone. BBR has been shown to significantly enhance glycemic control through various synergistic mechanisms when used in combination with pioglitazone and metformin (Adil et al., 2017; Lyu et al., 2019). Furthermore, we conducted a more detailed subgroup analysis based on the combination regimens of BBR and found that BBR combined with Met, SU, and DPP-4 inhibitors exhibited a synergistic effect. However, its combination with insulin exhibited improvements in HbA1c comparable to insulin monotherapy. Additionally, our subgroup analysis revealed that BBR combined with SU may have an effect on FPG comparable to that of SU alone. Singh et al. (2020) found in *in vitro* experiments that when BBR is co-incubated with SU, it may interfere with the metabolism of SU, potentially weakening its hypoglycemic effect to some extent. We also observed that the combination of BBR with insulin for improving 2hPBG, as well as BBR combined with Met for improving Fins, did not exhibit a notable synergistic effect compared to monotherapy. This could be related to the alteration of the composition of certain gut microbiota by BBR, which in turn affects the pharmacokinetics and therapeutic efficacy of the co-medication (Zhang et al., 2019; Laia et al., 2022). However, due to the limited number of included studies and the high heterogeneity in subgroup analyses, these conclusions remain uncertain. Therefore, future research should further explore the mechanisms of interaction between BBR and other hypoglycemic agents and validate these findings through clinical trials, to provide more precise therapeutic guidance for clinical practice.

In addition, in terms of improving insulin levels and lipid profiles (LDL-C, HDL-C, TC, and TG), we found that low-dose BBR (≤0.9 g/day) may have better effects than higher doses (>0.9 g/day). An animal study by Guo et al. (2011) indicated that high-dose BBR (300 mg/kg/day) significantly inhibited liver CYP enzyme activity during a 14-day dosing period, which might influence the effect of combined medication to some extent. In contrast, low-dose BBR (10–100 mg/kg/day) over the same period could regulate insulin and lipid metabolism more effectively without interfering with drug metabolism. Therefore, when considering the use of BBR in combination with hypoglycemic agents, determining the optimal dosage range to optimize therapeutic efficacy is particularly important.

Inflammatory biomarkers play a significant role in the pathogenesis of T2DM. They are closely associated with disease progression, the development of complications (Galantini et al., 2022; Du et al., 2023; Arif et al., 2024), and even increased mortality (Zhang J. et al., 2024). BBR exerts anti-inflammatory effects by inhibiting the NF-κB signaling pathway through an AMPK-dependent mechanism, thereby reducing the expression of pro-inflammatory cytokines (Yao et al., 2015). A recent umbrella meta-analysis (Nazari et al., 2024) showed that BBR supplementation effectively improves inflammatory markers in adults. We found that both BBR monotherapy and combination therapy significantly improved inflammatory markers, including reductions in CRP, IL-6, and TNF-α levels, further confirming the potential value of BBR in the comprehensive management of T2DM.

In this study, we observed significant statistical heterogeneity, which may be attributed to multiple factors, including the publication year of the studies, differences in baseline characteristics of patients, and genetic predispositions that may affect drug metabolism and therapeutic response. Additionally, different BBR combination regimens and treatment durations might also lead to inconsistent therapeutic effects. To explore the sources of heterogeneity in depth, subgroup analyses and meta-regression were conducted. Furthermore, we conducted sensitivity analyses to exclude studies with significant impacts on overall heterogeneity. However, considerable heterogeneity persisted in the glucose metabolism and lipid metabolism outcomes of BBR monotherapy and combination therapy with hypoglycemic agents. Through meta-regression and subgroup analyses, we were unable to identify specific sources of heterogeneity. We speculated that factors such as BMI, pancreatic function, differences in measurement times, the dosage of co-medication, and the dosage ratio of BBR may contribute to the high heterogeneity. Some studies did not specify the disease course of T2DM in the subjects or whether they received dietary and exercise interventions, which we believe may also contribute to the high heterogeneity. Moreover, the methodological limitations of the included studies, such as the lack of blinding and allocation concealment, may have contributed to the heterogeneity. Furthermore, we believed that there were interactions among factors contributing to heterogeneity. First, the limitations of study methodology may vary across research conducted in different years; earlier studies may have less rigorous designs, potentially introducing greater bias in results, whereas study designs may have improved over time. This interaction between methodological limitations and publication year could lead to discrepancies, influencing the overall assessment of BBR efficacy. Second, the timing of outcome measurements can affect the assessment of BBR’s efficacy in combination therapy. For instance, certain hypoglycemic agents have an optimal window of action within a specific period after treatment initiation; measurements taken outside this window may not accurately reflect the drug’s true effect. Additionally, the dosage of co-administered drugs and the dosage ratio of BBR to these drugs may influence both efficacy and side effects, and this impact may vary depending on the timing of the measurement. At last, obesity is a common comorbidity of T2DM. Patients with lower BMI often have a shorter T2DM course and may respond better to BBR. This interaction may introduce variability in overall results. From a multifactorial perspective, patients with higher BMI typically have a longer disease course and may require higher dosages or different dosage ratios in combination therapy to achieve optimal effects. Furthermore, these patients may need a longer time to exhibit treatment effects, and the timing of measurements during this period could influence efficacy assessment, contributing to overall heterogeneity. Future studies should further explore how these factors jointly influence outcomes and focus on controlling these variables to reduce heterogeneity.

Regarding safety, a total of 16 studies thoroughly evaluated adverse events. Typical side effects of BBR, such as gastrointestinal discomfort and hypoglycemia, can be anticipated due to its pharmacological actions and mechanisms. These side effects typically resolve spontaneously during the study period. In addition, no serious adverse drug reactions were observed. Hence, the appropriate use of BBR in treating T2DM is deemed safe. We performed funnel plots and Egger’s tests for all outcomes, and for those with potential publication bias, the trim-and-fill method was used to adjust the results. The robustness of the overall findings was confirmed. Although the effect size for Fins in BBR monotherapy was reversed after trimming and filling, overall, the results of our meta-analysis are considered reliable.

## 5 Strengths

This study has several strengths. This study conducted a comprehensive and systematic meta-analysis of BBR treatment for T2DM based on the use of BBR. Particularly, we performed in-depth subgroup analyses based on combination regimens to provide references for clinical treatment. To ensure robust and up-to-date findings, we established stringent inclusion criteria and employed meticulous search methods. Unlike previous meta-analyses (Guo et al., 2021), we excluded studies utilizing BBR fruit or Coptis extracts as interventions (Sanjari et al., 2020; Tahmasebi et al., 2019), as these dried root extracts contain additional alkaloids apart from BBR, potentially introducing bias to the outcomes. Additionally, unlike previous meta-analyses (Xie et al., 2022; Dong et al., 2012; Liang et al., 2019), our study incorporated analyses of lipid profiles, insulin levels and inflammatory markers, striving to present a thorough understanding of the effects of BBR on T2DM patients.

## 6 Limitations

Inevitably, certain limitations are presented in our study: First, our research is subject to geographical constraints, as the data predominantly derive from Asian populations. This may limit the broader generalizability of the findings to regions with genetic, lifestyle, and environmental differences from Asia. Therefore, we emphasize that when applying our results to different regions and countries, it is crucial to consider local medical facilities, patient compliance, and potential drug interactions. Second, due to the limited number of studies, certain parameters could not be subject to subgroup analysis, limiting the in-depth analysis of variables that might affect the efficacy of BBR. Third, despite conducting subgroup analyses and meta-regression, there remains significant heterogeneity in the study results, with the sources of some of this heterogeneity still not clearly identified. Hence, future studies should incorporate a more diverse geographical range, a broader array of literature resources, and more sophisticated methodological designs, in a bid to provide more robust evidence for the use of BBR in the treatment of T2DM.

## 7 Conclusion

Our study findings demonstrated the therapeutic potential of BBR in the management of T2DM, both as a monotherapy and in combination with conventional hypoglycemic agents. Nonetheless, the mechanisms of BBR in combination with other hypoglycemic agents still need to be further elucidated. Future research should adopt more diverse study designs and take into account factors such as the dosage of BBR, combination medications, treatment duration, and individual patient differences that may influence therapeutic outcomes. This will provide more precise guidance for clinical practice and help optimize treatment regimens for patients with T2DM.
