# Supplementary material for: Effects of administering berberine alone or in combination on type 2 diabetes mellitus: a systematic review and meta-analysis
Source: Front Pharmacol. 2024 Nov 21;15:1455534. doi: 10.3389/fphar.2024.1455534 (PMC11617981; doi:10.3389/fphar.2024.1455534)
Supplement: Supplementary file 2 [file Table3.docx]

**Jadad Scores for 50 studies**

|  | Randomization | Allocation hiding | Blinding | Withdrawais and Dropouts | Total |
| --- | --- | --- | --- | --- | --- |
| Rong et al. 1997 | 1 | 1 | 0 | 1 | 3 |
| Zhang et al. 2000 | 1 | 1 | 0 | 1 | 3 |
| Gao et al. 2002 | 1 | 1 | 0 | 1 | 3 |
| Liu 2004 | 1 | 1 | 0 | 1 | 3 |
| Guo et al. 2006 | 1 | 1 | 0 | 1 | 3 |
| Li et al. 2007 | 1 | 1 | 0 | 1 | 3 |
| Li 2008 | 1 | 1 | 0 | 1 | 3 |
| Li et al. 2008 | 2 | 1 | 0 | 1 | 4 |
| Yin et al. 2008 | 1 | 1 | 0 | 1 | 3 |
| Zhu et al. 2009 | 1 | 1 | 0 | 1 | 3 |
| Sheng et al. 2010 | 1 | 1 | 0 | 1 | 3 |
| Zhang 2010 | 1 | 1 | 0 | 1 | 3 |
| Meng et al. 2011 | 2 | 1 | 0 | 1 | 4 |
| Xiang et al. 2011 | 1 | 1 | 0 | 1 | 3 |
| Yin et al. 2011 | 1 | 1 | 0 | 1 | 3 |
| Zhang et al. 2011 | 1 | 1 | 0 | 1 | 3 |
| Liu 2012 | 1 | 1 | 0 | 1 | 3 |
| Wang et al. 2012 | 1 | 1 | 0 | 1 | 3 |
| Xue et al. 2012 | 1 | 1 | 0 | 1 | 3 |
| Zhang et al. 2012 | 1 | 1 | 0 | 1 | 3 |
| Zhou et al. 2012 | 1 | 1 | 0 | 1 | 3 |
| Shu 2014 | 2 | 1 | 0 | 1 | 4 |
| Yao 2015 | 0 | 1 | 0 | 1 | 2 |
| Yu 2015 | 1 | 1 | 0 | 1 | 3 |
| Zhu et al. 2015 | 2 | 1 | 0 | 1 | 4 |
| Du 2016 | 2 | 1 | 0 | 1 | 4 |
| Meng 2016 | 1 | 1 | 0 | 1 | 3 |
| Sun 2016 | 1 | 1 | 0 | 1 | 3 |
| Li 2017 | 1 | 1 | 0 | 1 | 3 |
| Sun 2017 | 2 | 1 | 0 | 1 | 4 |
| Xing 2017 | 2 | 1 | 0 | 1 | 4 |
| Wu 2017 | 1 | 1 | 1 | 1 | 4 |
| Zhang 2017 | 2 | 2 | 1 | 1 | 6 |
| Yao et al. 2018 | 1 | 1 | 0 | 1 | 3 |
| Fan et al. 2018 | 2 | 1 | 0 | 1 | 4 |
| Huang et al. 2018 | 1 | 1 | 0 | 1 | 3 |
| Rashidi et al. 2018 | 2 | 2 | 1 | 1 | 6 |
| Jiang et al. 2019 | 1 | 1 | 0 | 1 | 3 |
| Yang et al. 2020 | 2 | 1 | 0 | 1 | 4 |
| Zhu et al. 2020 | 2 | 2 | 2 | 1 | 7 |
| Zhang et al. 2020 | 2 | 2 | 2 | 1 | 7 |
| Chen et al. 2021 | 2 | 1 | 0 | 1 | 4 |
| Chen 2021 | 1 | 1 | 0 | 1 | 3 |
| Chen et al. 2021(b) | 2 | 1 | 0 | 1 | 4 |
| Ye 2021 | 1 | 1 | 0 | 1 | 3 |
| Wang et al. 2022 | 1 | 1 | 0 | 1 | 3 |
| Yu 2022 | 1 | 1 | 0 | 1 | 3 |
| Chen et al. 2023 | 2 | 1 | 1 | 1 | 5 |
| Lu 2023 | 2 | 1 | 0 | 1 | 4 |
| Yang et al. 2023 | 2 | 1 | 0 | 1 | 4 |

one used admission time to randomize the number of participants with high risk.
