# Supplementary material for: Effects of administering berberine alone or in combination on type 2 diabetes mellitus: a systematic review and meta-analysis
Source: Front Pharmacol. 2024 Nov 21;15:1455534. doi: 10.3389/fphar.2024.1455534 (PMC11617981; doi:10.3389/fphar.2024.1455534)

# Egger’s test(n≥10):

| Outcome | Number  of  studies | Egger’s test  *p* value | Trim and Fill | | Robustness |
| --- | --- | --- | --- | --- | --- |
|  |  |  | Before | After |  |
| 3.4.1.1 FPG | 17 | *P* = 0.554 |  |  | **√** |
| 3.4.1.3 HbA1c | 11 | *P* = 0.700 |  |  | **√** |
| 3.4.1.8 TC | 11 | *P* = 0.989 |  |  | **√** |
| 3.4.1.9 TG | 12 | *P* = 0.592 |  |  | **√** |
| 3.4.2.1 FPG | 30 | *P* = 0.261 |  |  | **√** |
| 3.4.2.2 2hPBG | 25 | *P* = 0.192 |  |  | **√** |
| 3.4.2.3 HbA1c | 25 | *P* = 0.007 | MD = -0.69%, 95% CI (-0.99, -0.39), p < 0.01 | MD = -0.88%, 95% CI (-1.14, -0.62), p < 0.01 | **√** |
| 3.4.2.4 Fins | 13 | *P* = 0.103 |  |  | **√** |
| 3.4.2.6 LDL-C | 11 | *P* = 0.261 |  |  | **√** |
| 3.4.2.7 HDL-C | 10 | *P* = 0.166 |  |  | **√** |
| 3.4.2.8 TC | 14 | *P* = 0.005 | MD = -0.61 mmol/L, 95% CI (-0.79, -0.43), p < 0.01 | MD = -0.61 mmol/L, 95% CI (-0.79, -0.43), p < 0.01  data unchanged | **√** |
| 3.4.2.9 TG | 15 | *P* = 0.016 | MD = -0.50 mmol/L, 95% CI (-0.61, -0.40), p < 0.01 | MD = -0.54 mmol/L, 95% CI (-0.64, -0.44), p < 0.01 | **√** |

# Funnel plots(n＜10):

| Outcome | Number  of  studies | Basically symmetrical | Trim and Fill | | Robustness |
| --- | --- | --- | --- | --- | --- |
|  |  |  | Before | After |  |
| 3.4.1.2 2hPBG | 8 | No | MD = -1.57 mmol/L, 95% CI (-2.71, -0.43), *p* < 0.01 | MD = -2.38 mmol/L, 95% CI (-3.38, -1.38), *p* < 0.01 | **√** |
| 3.4.1.4 Fins | 9 | No | SMD = -0.30, 95% CI (-0.71, 0.10), p = 0.136 | SMD = -0.459, 95% CI (-0.84, -0.07), p = 0.019 ＜ 0.05 | **×** |
| 3.4.1.5 HOMA-IR | 7 | Yes |  |  | **√** |
| 3.4.1.6 LDL-C | 9 | Yes |  |  | **√** |
| 3.4.1.7 HDL-C | 7 | Yes |  |  | **√** |
| 3.4.2.5 HOMA-IR | 6 | No | (SMD = -1.04, 95% CI (-1.81, -0.28), p＜ 0.01) | (SMD = -1.34, 95% CI (-2.05, -0.64), p ＜ 0.01) | **√** |
| 3.4.2.10 CRP | 9 | Yes |  |  | **√** |
| 3.4.2.10 1L-6 | 9 | Yes |  |  | **√** |
| 3.4.2.10 TNF-α | 8 | No | SMD = -1.58, 95% CI (-2.49, -0.68), p ＜ 0.01 | SMD = -1.77, 95% CI (-2.71, -0.82), p ＜ 0.01 | **√** |


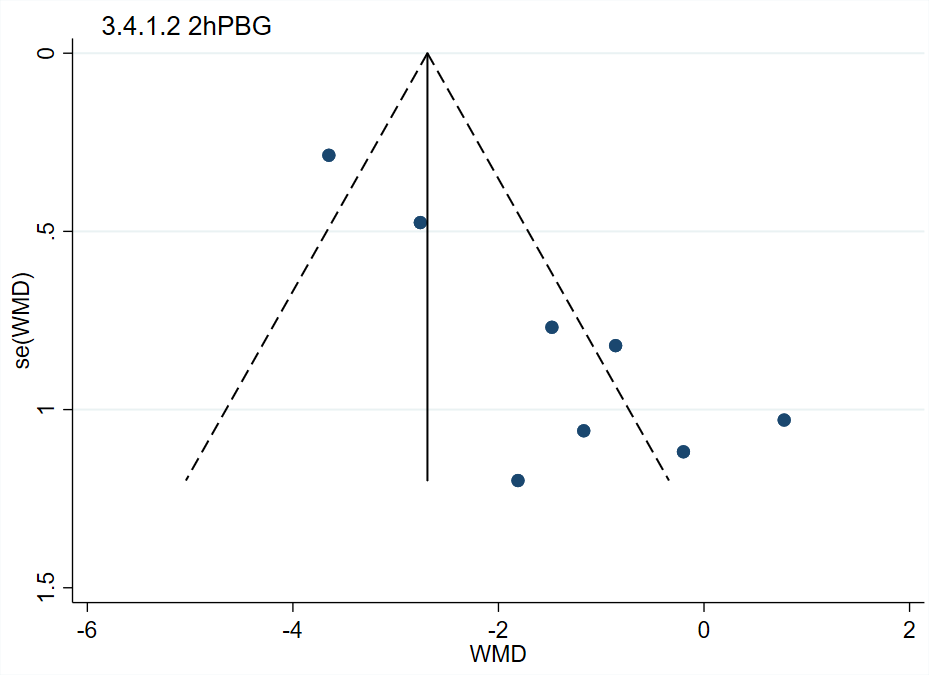

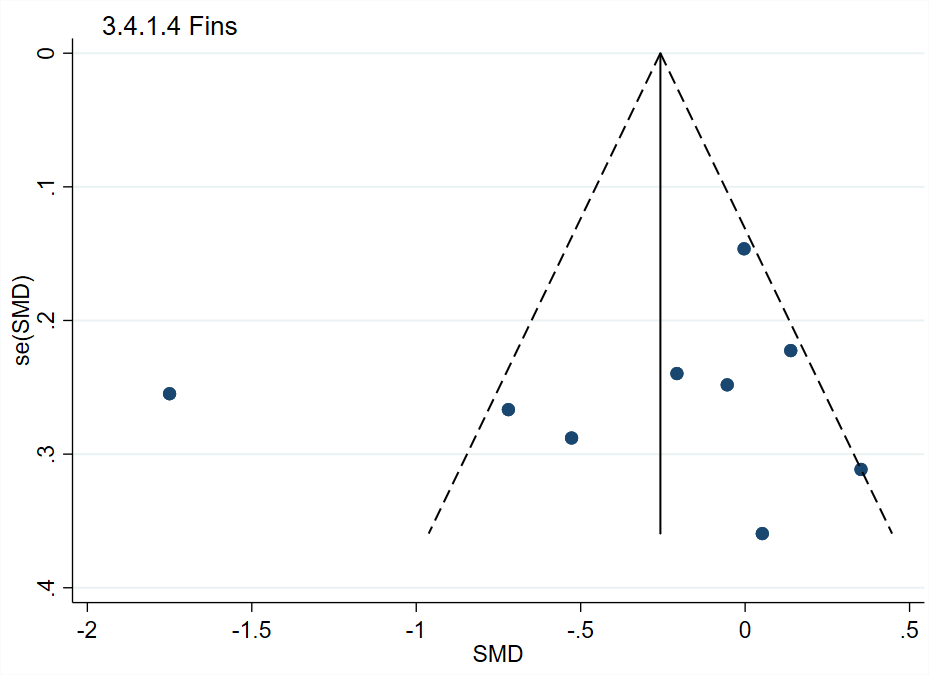

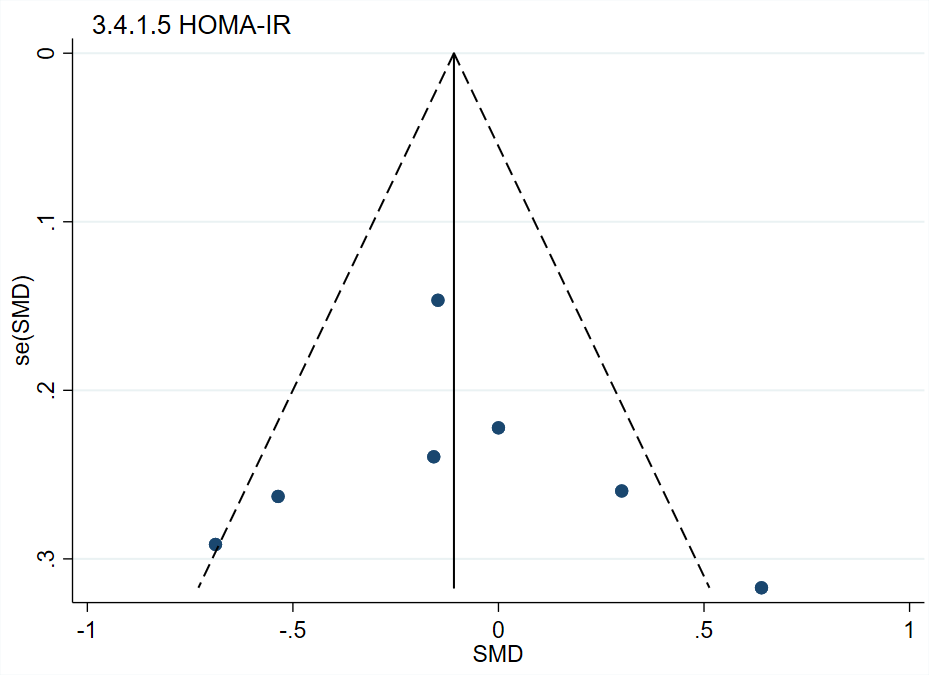


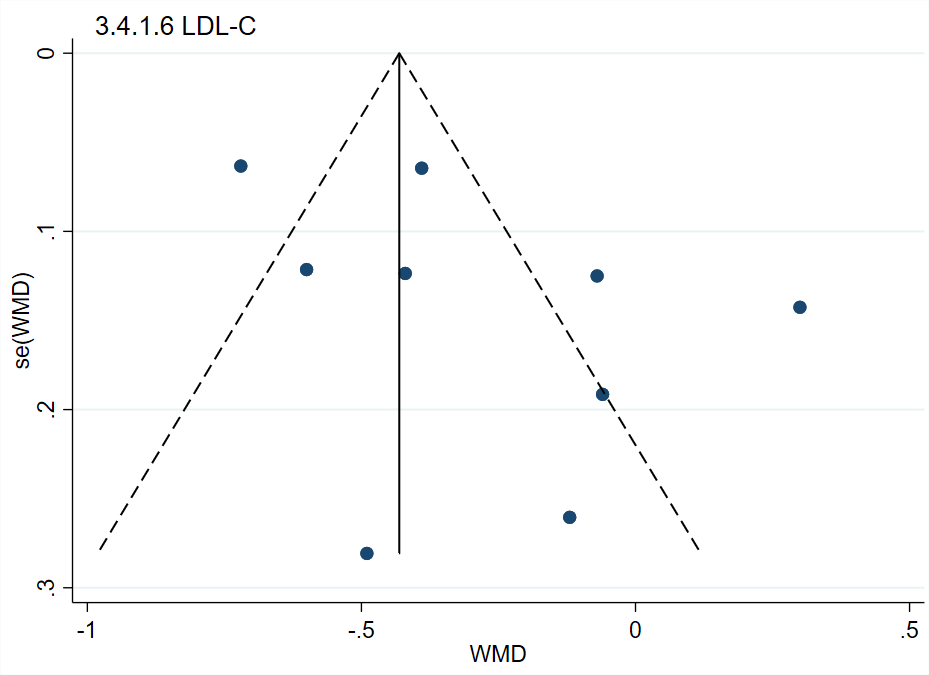


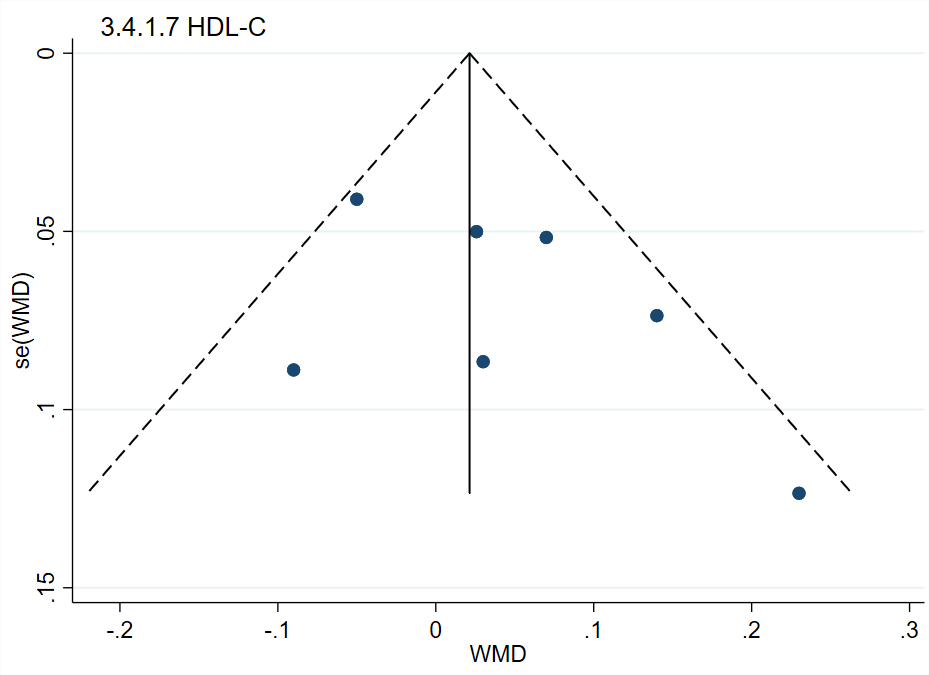


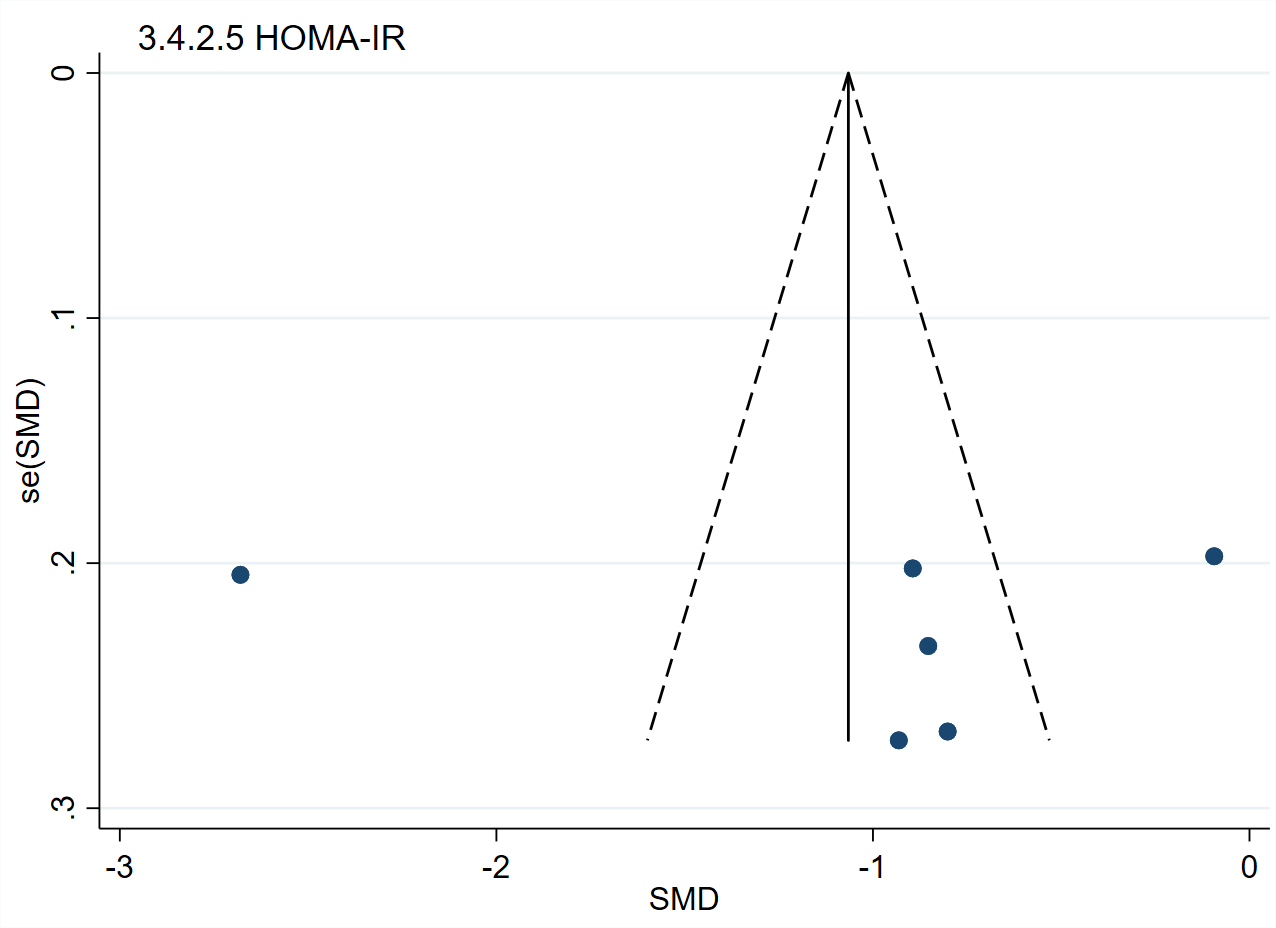


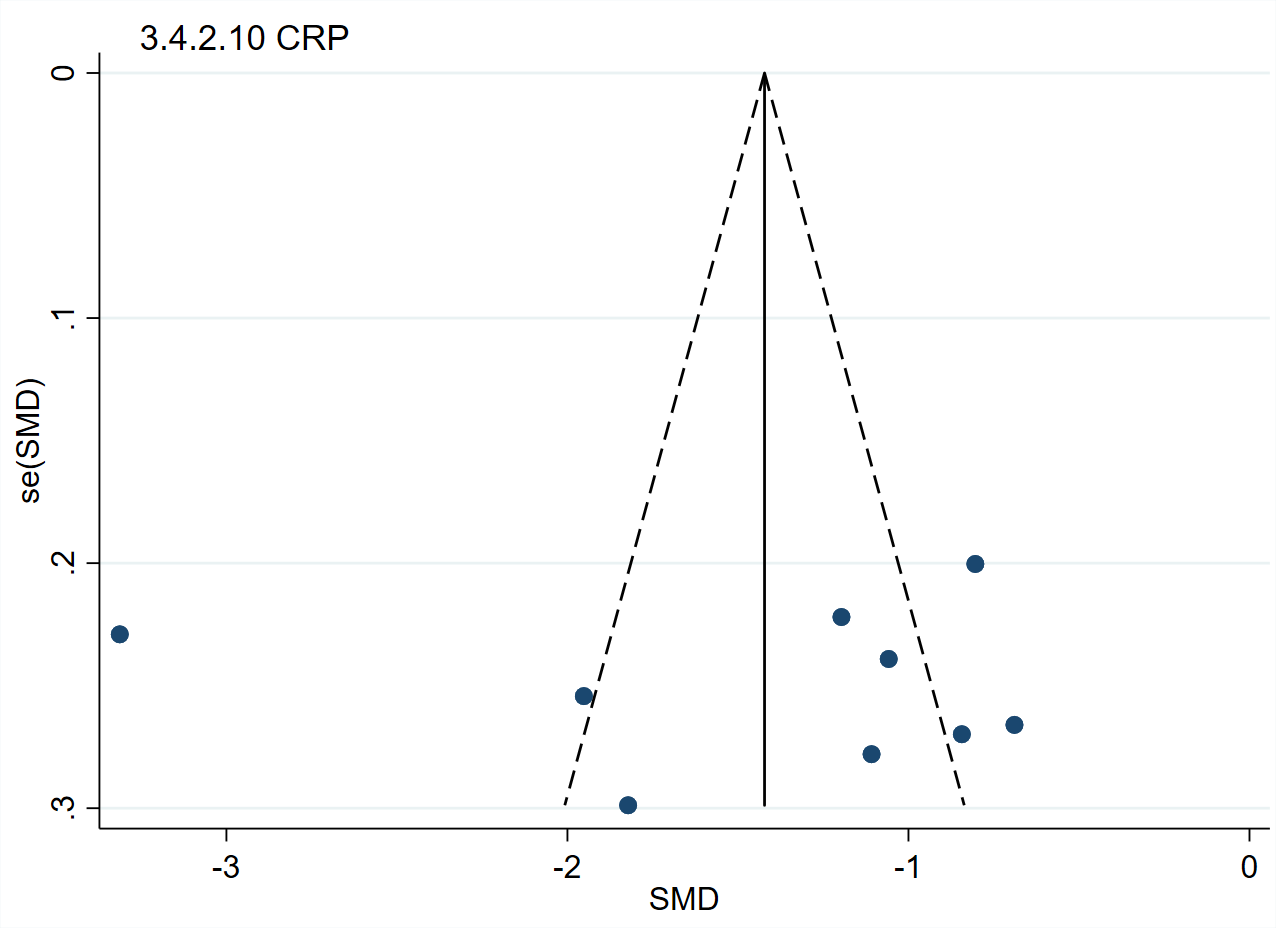


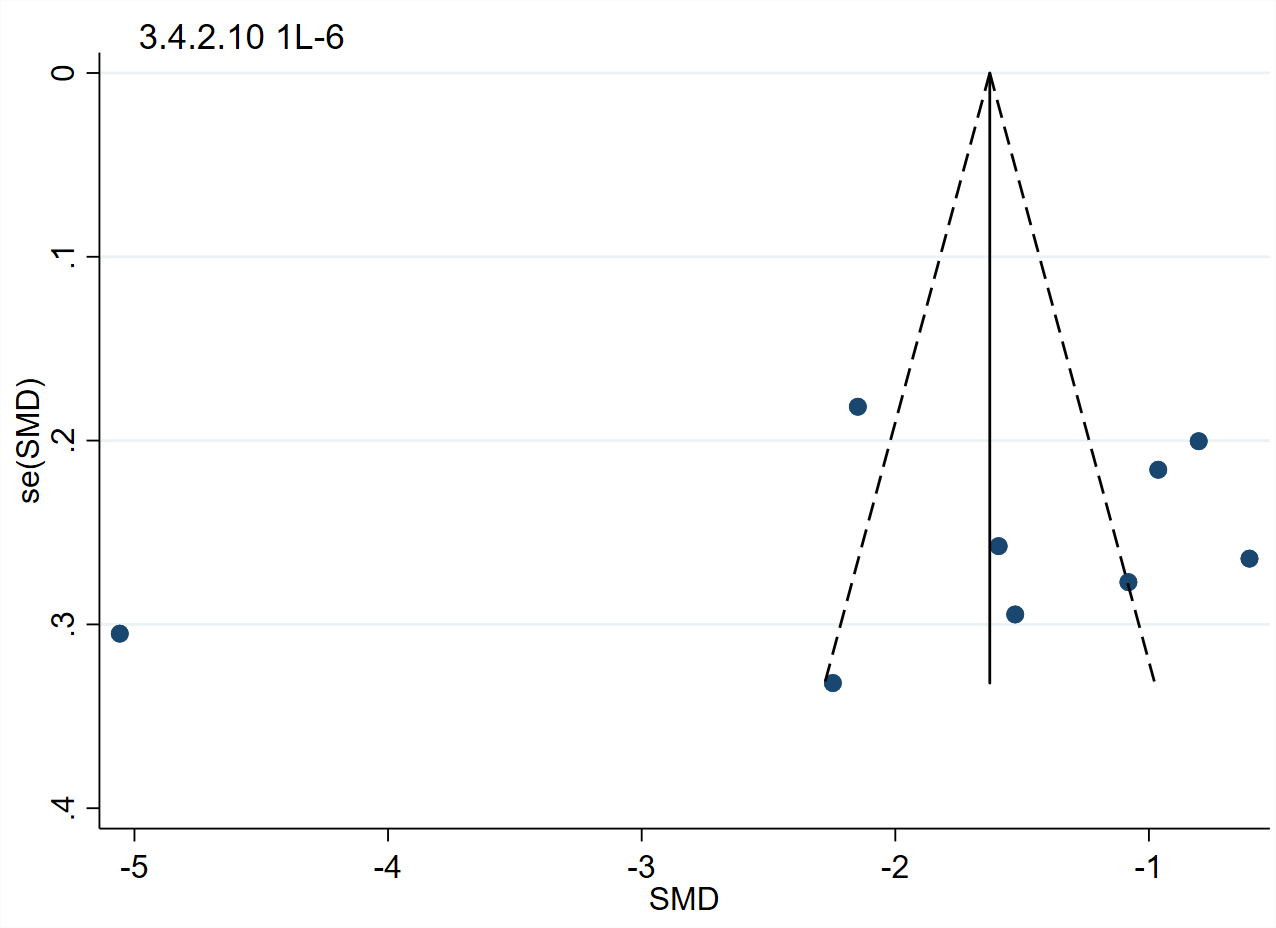


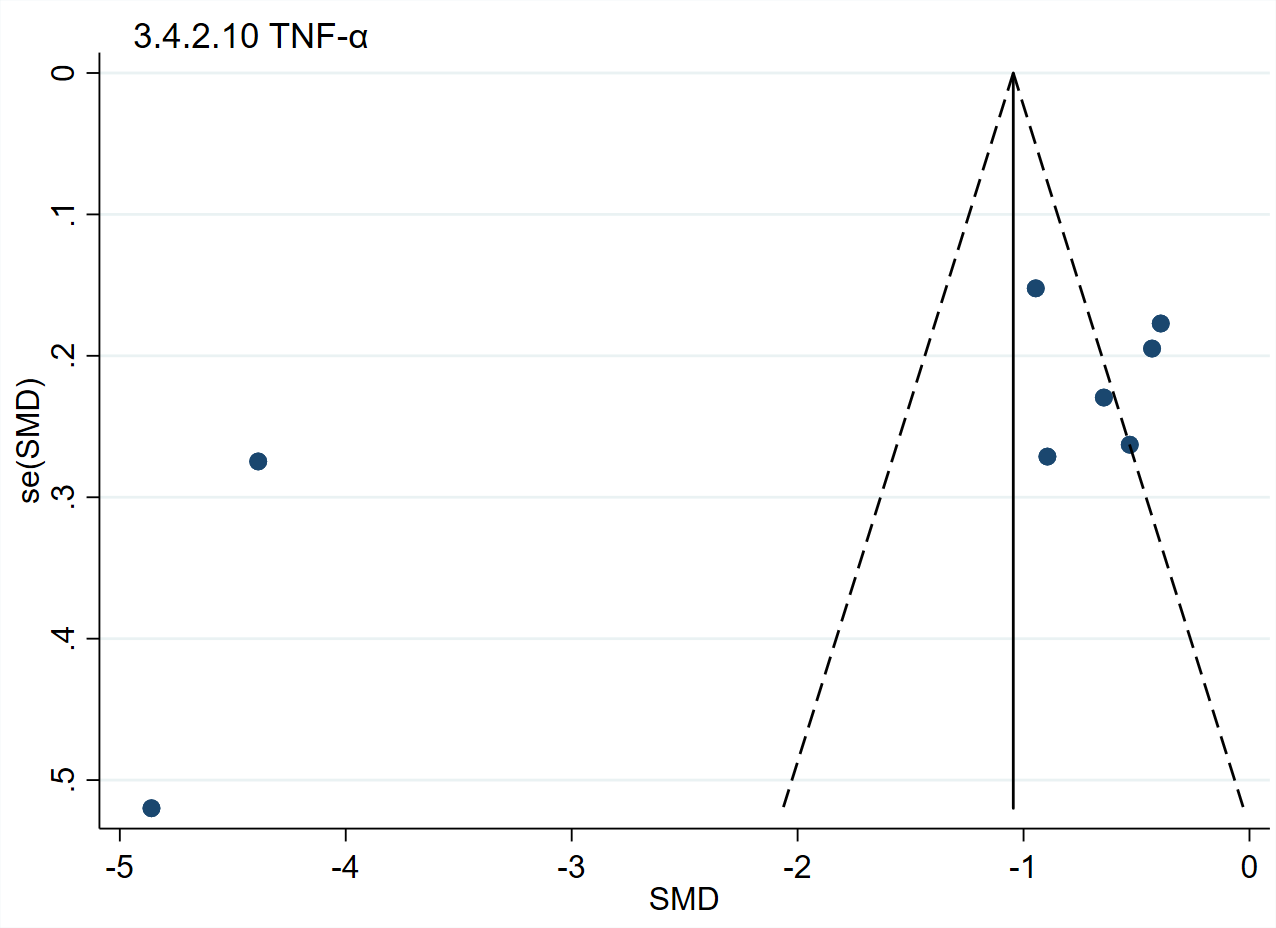

Supplement: Supplementary file 4 [file DataSheet1.docx]
